# Genomic and pathogenicity islands of *Listeria monocytogenes*—overview of selected aspects

**DOI:** 10.3389/fmolb.2023.1161486

**Published:** 2023-06-14

**Authors:** Natalia Wiktorczyk-Kapischke, Krzysztof Skowron, Ewa Wałecka-Zacharska

**Affiliations:** ^1^ Department of Microbiology, Ludwik Rydygier Collegium Medicum in Bydgoszcz, Nicolaus Copernicus University, Toruń, Poland; ^2^ Department of Food Hygiene and Consumer Health, Wrocław University of Environmental and Life Sciences, Wrocław, Poland

**Keywords:** *Listeria monocytogenes*, virulence, pathogenicity island (PAI), genomic island (GEI), genes, outbreaks, whole genome sequencing (WGS)

## Abstract

*Listeria monocytogenes* causes listeriosis, a disease characterized by a high mortality rate (up to 30%). Since the pathogen is highly tolerant to changing conditions (high and low temperature, wide pH range, low availability of nutrients), it is widespread in the environment, e.g., water, soil, or food. *L. monocytogenes* possess a number of genes that determine its high virulence potential, i.e., genes involved in the intracellular cycle (e.g., *prfA*, *hly*, *plcA*, *plcB*, *inlA*, *inlB*), response to stress conditions (e.g., *sigB*, *gadA*, *caspD*, *clpB*, *lmo1138*), biofilm formation (e.g., *agr, luxS*), or resistance to disinfectants (e.g., *emrELm*, *bcrABC*, *mdrL*). Some genes are organized into genomic and pathogenicity islands. The islands LIPI-1 and LIPI-3 contain genes related to the infectious life cycle and survival in the food processing environment, while LGI-1 and LGI-2 potentially ensure survival and durability in the production environment. Researchers constantly have been searching for new genes determining the virulence of *L. monocytogenes*. Understanding the virulence potential of *L. monocytogenes* is an important element of public health protection, as highly pathogenic strains may be associated with outbreaks and the severity of listeriosis. This review summarizes the selected aspects of *L. monocytogenes* genomic and pathogenicity islands, and the importance of whole genome sequencing for epidemiological purposes.

## Introduction

### General characteristics of *Listeria monocytogenes*

*L. monocytogenes* are Gram-positive, non-spore-forming, relatively anaerobic rods (Gandhi and Chikindas, 2007). *L. monocytogenes* easily adapt to environmental conditions (Muskalska and Szymczak, 2015), can grow in a wide range of temperatures (0°C–45°C), pH (4.3–9.6), tolerate high salt concentrations (up to 10.0% NaCl) and low water activity (A_w_ to 0.90) (Gandhi and Chikindas, 2007; Wiktorczyk-Kapischke et al., 2021). Adaptation to unfavorable environmental conditions is associated with the expression of many genes (Begley et al., 2005; Ryan et al., 2009; Kocaman and Sarimehmetoğlu, 2016; Wiktorczyk-Kapischke et al., 2021). Response to stressful conditions ensure genes localized on SSI-1 (stress survival islet-1): acid stress, osmotic stress, bile stress in the stomach (Ryan et al., 2010) and SSI-2 (stress survival islet-2): alkaline and oxidative stress (Harter et al., 2017). Table 1 presents a summary of the most important genes involved in pathogenesis and adaptation to stress.

**TABLE 1:** Genes and proteins involved in virulence and stress adaptation in *L. monocytogenes.*

Participation in	Site/Function	Gene	Protein	References
Pathogenesis/Virulence	LIPI-1 (involved in the intracellular infection cycle of *L. monocytogenes*)	*prfA*	Positive regulatory factor A (PrfA)	Vázquez-Boland et al. (2001a)
		*plcA*	Phospholipase A (PlcA)	
		*hly*	Listeriolysin O (LLO, pore-forming toxin)	
		*mpl*	Metalloprotease (Mpl)	
		*actA*	Actin assembly-inducing protein (ActA)	
		*plcB*	Phospholipase B (PlcB)	
	locus InlA-InlB (involvement in adhesion)	*inlA*	Internalin A (InlA)	
		*inlB*	Internalin B (InlB)	
	LIPI-3 (operon coding LLS - bacteriocin and hemolytic cytotoxic factor)	*llsA*	Listeriolysin L (LLS)	Cotter et al. (2008)
		*llsG*		
		*llsH*		
		*llsX*		
		*llsB*		
		*llsY*		
		*llsD*		
		*llsP*		
	LIPI-4 (infections of the central nervous system and placenta)	*lm4b_02324*	maltose-6′-P-glucosidase	Maury et al. (2016)
		*lm4b_02325*	transcriptional antiterminator	
		*lm4b_02326*	uncharacterized protein associated to PTS systems	
		*lm4b_02327*	membrane permease EIIA	
		*lm4b_02328*	membrane permease EIIB	
		*lm4b_02329*	membrane permease EIIC	
Stress adaptation/tolerance	LGI-1 (virulence, resistance to antimicrobial substances, and stress factors)*	*virB1*	cell wall-associated hydrolases (invasion-associated proteins)	Gilmour et al. (2010)
		*tadG*	Flp pilus assembly protein TadG	
		*cadA*	cation-transporting ATPase, P1-type	
		*virB11, cpaF, tadA*	Flp pilus assembly protein TadB	
		*erm*	putative cation and cationic drug efflux transporter	
		*virB6, trbL*	Type IV secretory pathway, TrbL components	
		*virB4, cpaB, cagE, trbE*	Type IV secretory pathway, VirB4 components	
		*ermELm*	efflux transporter (resistance to benzalkonium chloride)	Kovacevic et al. (2016)
	LGI-2*	*arsR1D2R2A2B1B2*	arsenic resistance cassette	Brires et al. (2011)
	SSI-1 (tolerance to acid, osmotic and bile stress in the stomach)	*lmo0444*	Predicted membrane proteins	Ryan et al. (2010)
		*lmo0464*	M-protein trans-acting positive regulator	
		*pva (lmo0446)*	penicillin V amidase	
		*gadD1 (lmo0447)*	Glutamate decarboxylases and related PLP-dependent proteins	
		*gadT1 (lmo0448)*	Amino acid transporters	
	SSI-2 (survival under alkaline and oxidative stress)	*lin0464*	Transcription factor LIN0464	Harter et al. (2017)
		*lin0465*	Pfpl (protease)	
	Osmotic stress	operon *gbuABC*	Gbu transporter	Mendum and Smith (2002)
		*betL*	Glycine betainę transporter (BetL)	
		operon *opuCABCD*	OpuC transporter	
	Heat stress (HSP—heat shock protein)	I class HSP: *groE, dnaK, dnaJ, groEL and groES*	GroE, DnaK, DnaJ, GroEL, GroES (chaperones)	Bucur et al. (2018)
		III class HSP: *clpB, clpC, clpP* and *clpE*	ClpB, ClpC, ClpP and ClpE	Nair et al. (2000)
	Cold stress	*cspA, cspB, cspD*	CspA, CspB, CspD	Schmid et al. (2009)
		*cap*	Cap	Barria et al. (2013)
		*ltrC*	Low-temperature requirement C protein	Bucur et al. (2018)
	Acid stress	*arcA*	Putative arginine deiminase	Ryan et al. (2009)
		*arcB*	Carbamoyltransferase	
		*arcC*	Carbamate kinase	
		*arcD*	Antiporter	
	Oxidative stress	*lmo1433*	Glutathione reductase	Kazmierczak et al. (2003)
Resistance to disinfectants	Mobile genetic elements	*brcABC*	Efflux pump BrcABC (resistance to benzalkonium chloride)	Elhanfi et al. (2010); Møretrø et al. (2017)
		*emrE*	Efflux pump ErmE (resistance to benzalkonium chloride)	Meier et al. (2017)
		*mdrL*	Efflux pump MdrL (resistance to benzalkonium chloride)	Yu et al. (2018)

LIPI- *Listeria* pathogenicity island; LGI, *Listeria* Genomic Island; SSI, Stress Survival Islet; * listed the most important; Csp - cold-shock protein; Cap–cold acclimatization protein. The italicised entries refer to gene names.

*L. monocytogenes* were divided into three evolutionary lineages, 14 serotypes, grouped into four serogroups (1/2a-3a, 1/2b-3b-7, 1/2c-3c, and 4b-4d-4e) (Doumith et al., 2004) (Table 2). In 2019, Yin et al. (2019) have described serotype 4h, HSL-II hybrid sublineage. The results reported by Maury et al. (2016) distinguished three categories of the most common clones of *L. monocytogenes*, e.g., CC1, CC2, CC4 and CC6—associated with infection; CC9 and CC12—food related clones; intermediate clones. Additionally, *L. monocytogenes*can be divided into four epidemic clones - ECI, ECII, ECIII and ECIV (Kathariou, 2003; Chen et al., 2007).

**TABLE 2:** Division into evolutionary lineages in *L. monocytogenes* serotypes (based on: Seeliger and Hőhne, 1979; Seeliger and Langer, 1989; Roberts et al., 2006; Ward et al., 2008; Orsi et al., 2011).

Evolutionary lineages	Serotype	Antigen O	Antigen H
**II**	1/2a	I, II, (III)	A, B
**I**	1/2b	I, II, (III)	A, B, C
**II**	1/2c	I, II, (III)	B, D
**II**	3a	II, (III), IV	A, B
**I**	3b	II, (III), IV, (XII), (XIII)	A, B, C
**II**	3c	II, (III), IV, (XII), (XIII)	B, D
**III**	4a	(III), (V), VII, IX	A, B, C
**III**	4ab	(III), V, VI, VII, IX, X	A, B, C
**I**	4b	(III), V, VI	A, B, C
**III**	4c	(III), V, VII	A, B, C
**I**	4d	(III), (V), VI, VII	A, B, C
**I**	4e	(III), V, VI, (VIII), (IX)	A, B, C
**I**	7	(III), XII, XIII	A, B, C

bracket—variables.

### Occurrence of *Listeria* monocytogenes

The ability to survive unfavorable conditions determines the ubiquitous nature of*L. monocytogenes* in the environment. The rods are isolated from, e.g., water, soil, sewage, rotting vegetation, and animal feed, as well as from various species of fish, birds, and mammals (Nightingale et al., 2005; Skowron et al., 2019; Liu et al., 2020; Al et al., 2022; Cavalcanti et al., 2022; Tahir et al., 2022). Food is the main source of *L. monocytogenes* for humans. This foodborne pathogen was isolated from variety of food, e.g., raw and smoked fish, meat products, unpasteurized milk products, as well as from ready-to-eat (RTE) products (European Food Safety Authority, 2018; European Centre for Disease Prevention and Control, 2019a; Skowron et al., 2019; Centers for Disease Control and Prevention, 2022a). Food of non-animal origin (FNAO) may also be a source of *L. monocytogenes* (EFSA Panel on Biological Hazards BIOHAZ Panel, 2013). FNAOs are products derived from plants and are an ingredient in almost every meal. FNAOs include fruits, vegetables, nuts and seeds, herbs, spices, or, mushrooms and algae (EFSA Panel on Biological Hazards BIOHAZ Panel, 2013). The first outbreak of listeriosis associated with FNAO was recorded in Boston (United States, 1979). The source of the rods was raw celery, tomatoes and lettuce (Ho, 1986). The feature of *L. monocytogenes*, which determines its presence in the food processing environment, is its resistance to disinfectants (Elhanafi et al., 2010; Ratani et al., 2012; Kovacevic et al., 2016; Meier et al., 2017; Møretrø et al., 2017; Yu et al., 2018) in *L. monocytogenes* (Table 1). Genes located on LGI-2 (*Listeria* Genomic Island 2) confer arsenic-cadmium resistance (Lee et al., 2013; Parsons et al., 2017; Haubert et al., 2019). High virulence and ubiquitous nature of *L. monocytogenes* may pose a relevant public health problem. Disinfectant resistance determines the presence of *L. monocytogenes* in the food processing environment, which can be a source of food contamination. Knowledge on the mechanisms conferring resistance and searching for new methods of *L. monocytogenes*eradication is indispensable to limit the spread of the rods and listeriosis outbreaks.

### Pathogenicity

*L. monocytogenes* is the etiological factor of listeriosis, characterized by a high mortality rate (up to 30%) (World Health Organization, 2018a). *L. monocytogenes* serotypes: 4b, 1/2b, and 1/2c (98% of documented cases) are most often responsible for listeriosis (Wiedmann et al., 1997; Liu et al., 2006). The most vulnerable to infection are elderly (over 65 years old), pregnant women, newborns and people with reduced immunity (cancer, diabetes, transplant, HIV (human immunodeficiency virus-infected, alcoholics) (Gandhi and Chikindas, 2007; World Health Organization, 2018a). *L. monocytogenes* has the ability to colonize the intestine and cross the blood-brain and placenta barriers (Kammoun et al., 2022). Genes localized on LIPI-1 (*Listeria* Pathogenicity Island 1) and the InlA-InlB locus (Vázquez-Boland et al., 2001a) are implicated in the infectious cycle of *L. monocytogenes*. Transmission of the microorganism to newborns may occur in the womb or in the infected birth canal during childbirth (Allerberger and Wagner, 2010). In the case of the central nervous system infection, as much as 50.0% of disease cases are fatal (Godziszewska et al., 2015). Genes of LIPI-4 participate in neuroinfection and fetal infection (Maury et al., 2016). Cases of listeriosis are reported most often in late summer and early fall (Lamont et al., 2011). First-line drugs in the listeriosis treatment include ampicillin or gentamicin (Temple and Nahata, 2000; Hof, 2004). Also rifampicin, vancomycin, linezolid and carbapenems (Goulet and Marchetti, 1996; Benes et al., 2002; Hof, 2004) or trimethoprim (in patients allergic to beta-lactams) are recommended (Benes et al., 2002; Hof, 2004). In recent years there has been an increase in antibiotic resistance in *L. monocytogenes* (Morvan et al., 2010). This phenomenon may contribute to therapeutic difficulties in the following years, especially in the case of multi-antibiotic-resistant strains.

In recent years, many foodborne outbreaks of listeriosis have been registered worldwide, e.g., in Republic of South Africa (ready-to-eat processed meat products, 2017–2018) (Smith et al., 2019), Australia (rockmelons, 2018) (World Health Organization, 2018b), and several epidemics in the United States, e.g., mexican-style cheese, 1985 (Beckers et al., 1987) or deli meats, 2020 (Centers for Disease Control and Prevention, 2021). The last documented epidemics of listeriosis was associated with Big Olaf’s ice cream. This outbreak included 23 cases of listeriosis (22 hospitalized, one death) (Centers for Disease Control and Prevention, 2022b). A number of listeriosis outbreaks linked to FNAO have been reported in the United States, e.g., raw broccoli and cauliflower (Simpson, 1996), cantaloupe (Centers for Disease Control and Prevention, 2011), celery (Gaul et al., 2013), caramel apples, (Centers for Disease Control and Prevention, 2015a). Also, two independent listeriosis outbreaks associated with packaged salads have been reported. One in 2016 involved 9 states and 19 cases (1 death) (Centers for Disease Control and Prevention, 2016d). The second, in 2022, covered eight states, 10 confirmed hospitalizations and 1 death (Centers for Disease Control and Prevention, 2022c). These data indicate the need for continuous monitoring of FNAO for the presence of *L. monocytogenes*.

The epidemiology of listeriosis infections associated with FNAO in the European Union varies. Between 2007 and 2011, the EU have reported on pre-packed mixed salad vegetables (England) (Little et al., 2010) and mixed salads (Greece) (Gillespie et al., 2010) contaminated with *L. monocytogenes*. Prior to 2007, cases of listeriosis associated with FNAO included: rice salad (Italy) (Salamina et al., 1996), salted mushrooms (Finland) (Junttila and Brander, 1989), vegetable rennet England (Kerr et al., 1988). Lately *L. monocytogenes* was detected in vegan cheeses and one vegetable pâté produced in France between April and December 2022. Five people were sick, including four pregnant women who gave birth prematurely. Contaminated products were distributed in Austria, Belgium, Germany, Italy, the Netherlands, Singapore, Spain, Switzerland, and the United Kingdom (https://ask-bioexpert.com/news/in-france-recall-of-various-jay-and-joy-vegan-products-due-to-listeria-monocytogenes/; https://www.foodsafetynews.com/2023/01/five-sick-in-french-listeria-outbreak-linked-to-cheese-alternative/; https://webgate.ec.europa.eu/rasff-window/screen/notification/591930). The presented statistics indicated the need to evaluate the presence of *L. monocytogenes* in FNAO products. Wartha et al. (2023a) have identified *L. monocytogenes* among 1.72% of FNAO (Germany) product samples. *L. monocytogenes* strains isolated from FNAO were resistant to benzylpenicillin, fosfomycin, and moxifloxacin (Wartha et al., 2023b). Continuously reported cases of listeriosis confirm the pathogenic nature of *L. monocytogenes* and the presence of rods in food products. Phenotypic and genetic evaluation of *L. monocytogenes* strains responsible for epidemics would be a valuable element in understanding the ecology of these rods. The search for new genetic determinants underlying virulence, antibiotic and disinfectant resistance is crucial for better understanding these pathogenic rods.

Knowledge of the pathogenic and adaptive nature of *L. monocytogenes*, especially strains isolated from food and the food industry, is a relevant aspect allowing reduction the of listeriosis cases number (Disson et al., 2021). It is also essential to understand the genetic basis of *L. monocytogenes* virulence in combination with phenotypic features (Stratakos et al., 2020; Lakicevic et al., 2022). More, genes involved in the stress response and adaptation to changing conditions also influence the pathogenic nature of *L. monocytogenes* (Wiktorczyk-Kapischke et al., 2021).

This review aims to characterize selected aspects of the genomic and pathogenicity islands in *L. monocytogenes*. We emphasize the importance of the Whole Genome Sequencing (WGS) technique for the identification of new genetic elements in *L. monocytogenes* and its use in epidemiological research. Knowledge of the virulence of *L. monocytogenes* could help prevent future listeriosis outbreaks through the appropriate control strategies selection.

## Pathogenicity and adaptation to stress conditions

### The infectious cycle

**FIGURE 1:**
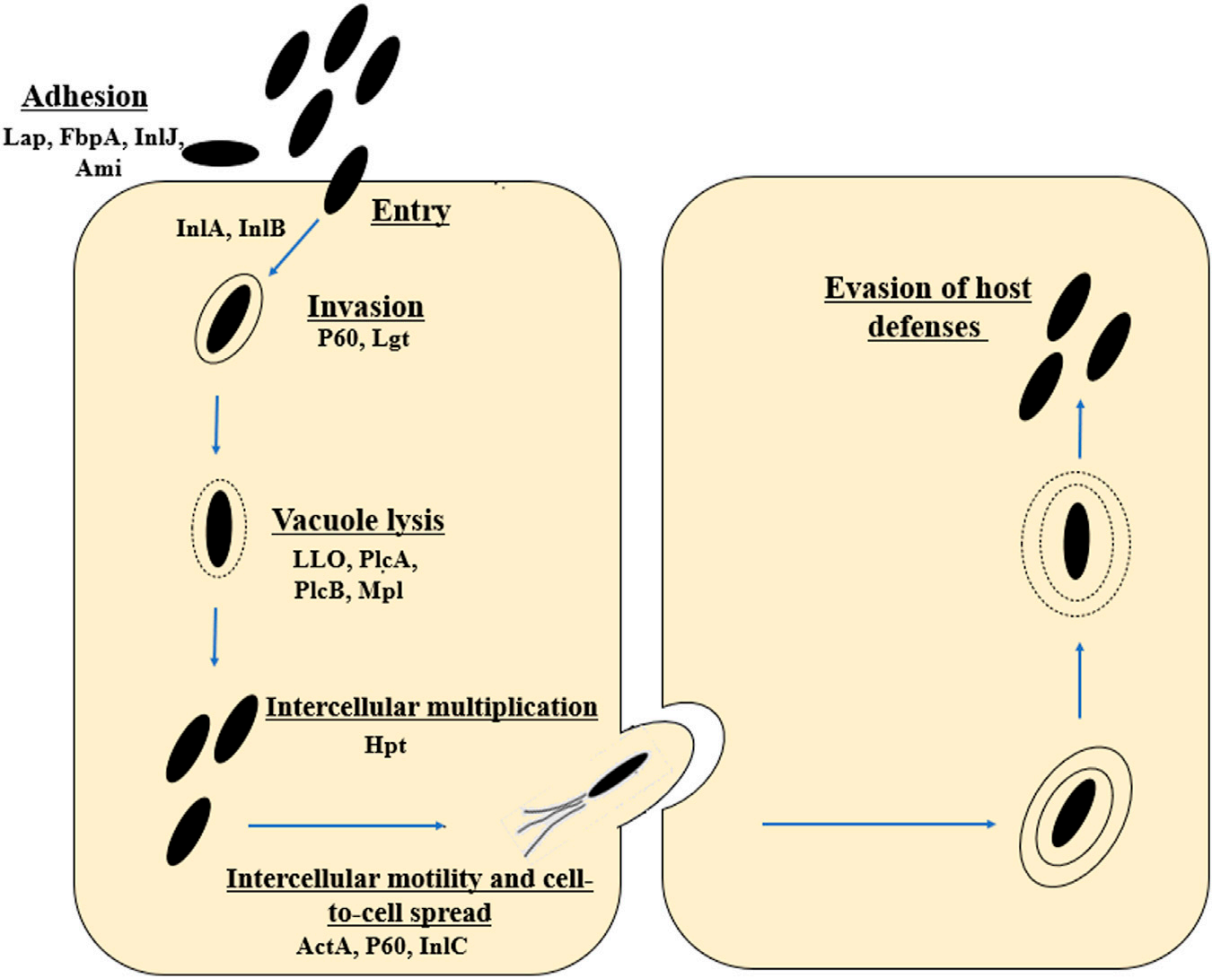
Stages of the life cycle of *L. monocytogenes* (virulence factors involved in each stage are included). The first stage includes adhesion (Lap, FbpA, inlJ, Ami, RecA), entry and internalization (InlA, InlB). Next stages are invasion (P60, Lgt), vacuole lysis (LLO, PlcA, PlcB, Mpl), intracellular proliferation (Hgt) and intracellular movement (ActA, P60, InlC). Lap, *Listeria* adhesion protein; FbpA, Fibronectin-binding protein; InlJ, interanlin J; Ami, autolysin amidase; InlA, internalin A; InlB, internalin B; Lgt, prolipoprotein diacylglyceryl transferase; LLO, Listeriolysin O; PlcA, secreted phosphatidylinositol-specific phospholipase C; PlcB, phospholipase C; Mpl, Metalloprotease; ActA, Actin assembly-inducing protein (according to: Southwick and Purich, 1996; Portnoy et al., 2002; Freitag et al., 2009; Ireton et al., 2021).

*L. monocytogenes* is an intracellular pathogen (Tilney and Portnoy, 1989). The mechanisms by which the rod penetrates and then multiplies in the host cells are still being intensively studied. *L. monocytogenes* uses a wide variety of virulence factors to promote host cell invasion (InlA, InlB), phagosome escape (LLO, PlcA and PlcB), rapid cytoplasmic replication (Hpt), and spread from cell to cell (ActA, InlC) (Hamon et al., 2006; Krypotou et al., 2019) (Figure 1). The main virulence factors essential for invasion include internalins (InlA and InIB) and listeriolysin O (LLO, encoded by the *hly* gene) (Phelps et al., 2018). The first stage of infection is adhesion to the surface of cells and internalization of their cytoplasmic membrane (Camejo et al., 2011). The Ami protein is responsible for the cleavage of the amide bond in the peptidoglycan. The Lap protein is an adhesin and murein hydrolase involved in the invasion of non-phagocytic cells (Ireton, 2007; Camejo et al., 2011). The FbpA protein, which enables fibronectin binding and protects *L. monocytogenes* from identification by the human immune system, also plays an important role at this stage (Ireton, 2007; Camejo et al., 2011). In *L. monocytogenes*, 27 proteins belonging to the internalin family (surface proteins) have been identified. In order to bind to the membrane surface, the rods utilize anchoring domains, particularly the LPXTG motif mediated by sortase A (Sabet et al., 2005). Internalin A exhibits strict cell tropism during its invasion of host cells, limited only to cells of epithelial origin (Bonazzi et al., 2009). The InlA is covalently attached to the bacterial cell wall by the LPXTG motif (Sabet et al., 2005; Pizarro-Cerdá and Cossart, 2006). InlA binds to E-cadherin, which interact with catenines, leading to the reorganization of the host cell actin cytoskeleton and phagocytosis (Camejo et al., 2011). The InlA/E-cadherin interaction is species-specific (Lecuit et al., 1999; Lecuit, et al., 2001). Researchers have reported premature stop codons (PMSCs) in the *inlA* gene of *L. monocytogenes* strains (Jonquieres et al., 1998; Olier et al., 2003; Jacquet et al., 2004; Handa-Miya et al., 2007). These mutants had a reduced ability to invade host cells (Nightingale et al., 2008). Internalin B uses three receptors, i.e., c—Met (transmembrane hepatocyte growth factor receptor - HGF), heparin, heparin sulphate proteoglycan (HPSG) and the qC1q—R glycoprotein (Bonazzi et al., 2009; Camejo et al., 2011). Met is a ubiquitous tyrosine kinase receptor that controls cell migration and growth during embryogenesis, tumor cell invasion and metastasis (Trusolino and Comoglio, 2002). InlB enables the invasion of cells of various types and origins (the c-Met receptor is expressed in a wide range of cells) (Ireton, 2007). As a result, *L. monocytogenes* invades the cell through phagocytosis (bacteria closed in the vacuole). Next, rods escape from the vacuole. The most important virulence factors at this stage include listeriolysin O (LLO), phospholipases A and B (PlcA and PlcB), metalloprotease (Mpl). LLO is a pore-forming toxin, enabling the lysis of the vacuole membrane and the entry into the host cytoplasm (Kayal and Charbit, 2006). PlcA, PlcB and Mpl participate in vacuole lysis, supporting the action of LLO (Camejo et al., 2011). PlcA, PlcB and Mpl participate in vacuole lysis, supporting the action of LLO (Camejo et al., 2011). Next stage is intercellular multiplication (Hpt), and spread to neighboring cells (ActA, InlC, P60) (Wuenscher et al., 1993). *L. monocytogenes* enters a neighboring cell with the formation of a secondary vacuole. Once released into the cytoplasm, the cycle is initiated anew (Ireton, 2007; Camejo et al., 2011) (Figure 1).

Other factors promoting the intracellular cycle are discussed later in this paper during LIPI-1description.

*L. monocytogenes* can survive and multiply in typical phagocytic cells but also attack and multiply in non-phagocytic cells (Lecuit, 2007; Seveau et al., 2007). The pathogen crosses the epithelial barrier by transcytosis invading the basal lamina propria (Nikitas et al., 2011). The bacterium reaches the mesenteric lymph nodes (MLN) through the lymphatic vessels and spreads to the liver and spleen via the lymph and blood. The bacteria can spread to secondary infection targets such as the central nervous system and the placenta. From the intestine, *L. monocytogenes* can also reach the liver via the hepatic portal vein (Melton-Witt et al., 2012). Next, *L. monocytogenes* can translocate into the gallbladder through the bile ducts. Due to extracellular replication in the bile ducts (Hardy et al., 2006; Eimerman, 2010) *L. monocytogenes* can be reintroduced into the gastrointestinal tract (Hardy et al., 2006).

In response to immune cells infection by *L. monocytogenes*, the host organism induces the production of many cytokines (Hansen et al., 2014). Induction of IFNα/ß and cytokines during *L. monocytogenes* infection results from the bacterial wall components recognition by Toll-like receptors (TLRs) and additional molecules of bacterial origin by intracellular TLR-independent mechanisms (Leber et al., 2008; Stockinger et al., 2009; Yang et al., 2010; Abdullah et al., 2012). The presence of *L. monocytogenes* DNA in the cytoplasm of host cells is a potent activator of the induction of IFN type I (Stetson and Medzhitov, 2006; Hansen et al., 2014). Moreover, listeriolysin O potentially stimulates IFN induction (Woodward et al., 2010; Burdette et al., 2011).

### Adaptaion to stress factors

**FIGURE 2:**
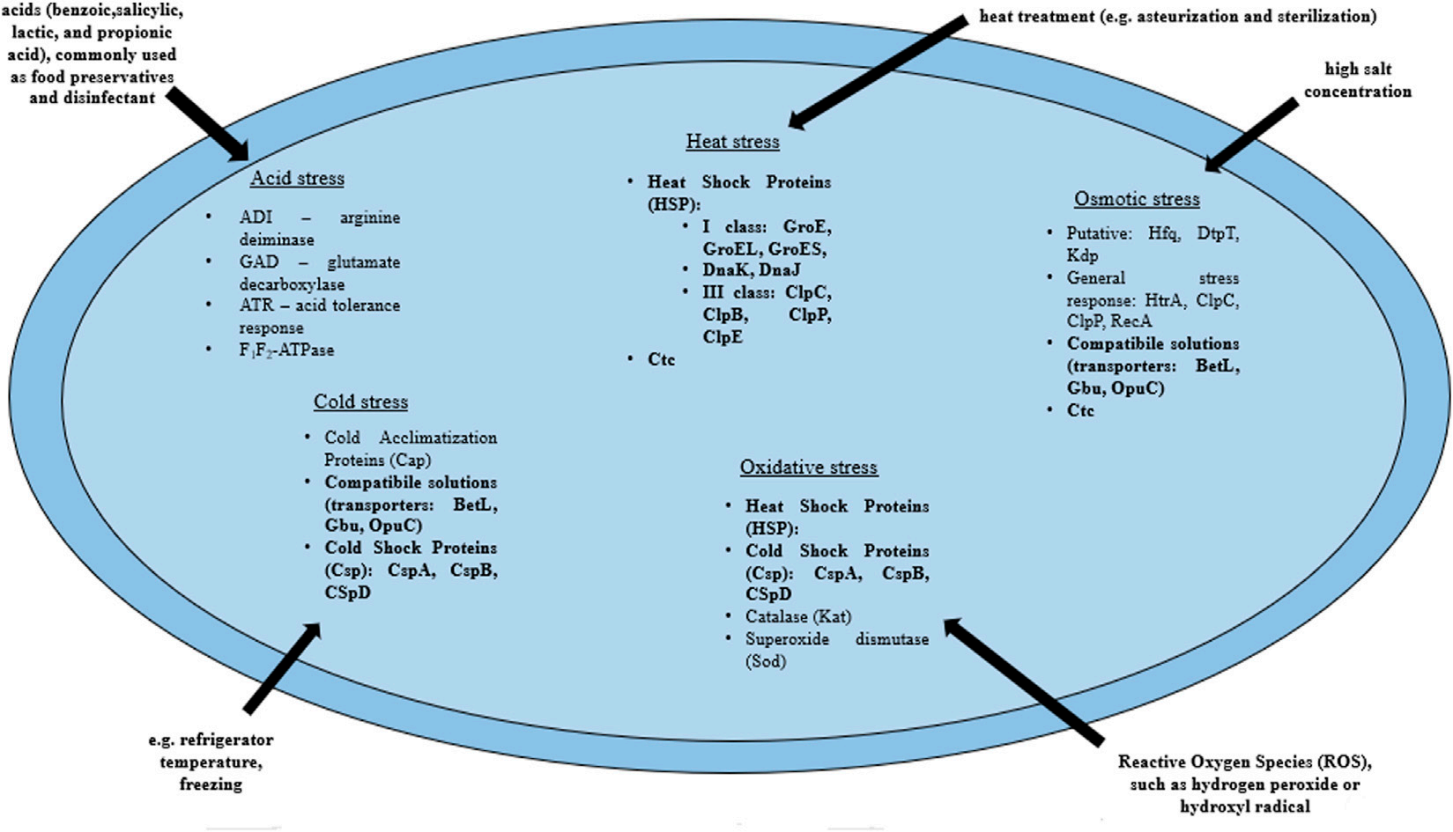
Mechanisms of the stress response in *L. monocytogenes* (bold print indicates the mechanisms involved in the response to several stressors). BetL, Glycine betaine transport system I; Gbu, Glycine betaine transport system II; OpuC, Carnitine transport system; ClpC, ATPase protein; ClpP, Serine protease; ClpB, Chaperone protein ClpB; ClpE, ATPase protein; GroES, GroE—Chaperone proteins which regulate HrcA posttranscriptionally; HtrA, Serine protease (according to: Wiktorczyk-Kapischke et al., 2021).

*L. monocytogenes* are present in many environments as the bacteria tolerate a wide range of variable conditions (Wiktorczyk-Kapischke et al., 2021), both during host invasion (Krypotou et al., 2019) and also in the food processing environment (O’Byrne and Karatzas, 2008). Some strains of *L. monocytogenes* are avirulent. On the other hand, some strains may increase virulence after exposure to environmental stress (Stratakos et al., 2020). Exposure to environmental stress influences cell morphology, antimicrobial resistance (AMR), pathogenicity and virulence of *L. monocytogenes* through the expression of a number of genes (Matereke and Okoh, 2020). More, sublethal stress may contribute to stress adaptation (resistance to higher levels of the same stress factor) or cross-resistance (resistance to other stress factors) (Faezi-Ghasemi and Kazemi, 2015). The pathogen has developed a number of mechanisms that allow adaptation to stressful conditions. The most frequently encountered adverse conditions by *L. monocytogenes* include: osmotic, heat, cold, acid, alkaline and nutrients stresses (Cotter et al., 2005; Giotis et al., 2010; Kocaman and Sarimehmetoğlu, 2016; Bucur et al., 2018; Manso et al., 2020). Stress response in *L. monocytogenes* involves many genes, followed by proteins that include general stress proteins and mechanisms related to adaptation to specific conditions (Wiktorczyk-Kapischke et al., 2021) (Table 1, Figure 2).

The response to osmotic stress relies on the compatible substances accumulation from the environment. This involves the respective transporters, i.e., BetL and Gbu (glycineabetaine transport) and OpuC (carnitine transport). In the absence of osmoprotectants in the environment, general stress proteins (Ryan et al., 2009) participate in the osmotic stress response. Heat shock protein (Hsp) synthesis begins in *L. monocytogenes* under high temperature conditions. Class I proteins act as chaperone intracellular proteins, and class III: ATP-dependent proteins with caseinolytic activity (Table 1). Also, GroEL and GroES proteins (regulation of basic cellular processes) and DnaK and DnaJ proteins (stabilization of unfolded proteins conformations) are involved in the heat stress response in *L. monocytogenes* (Nair et al., 2000; Bucur et al., 2018). In response to a drop in temperature, *L. monocytogenes* begins to synthesize cold shock proteins (Csp). Csp are molecular chaperone proteins that enable replication, transcription and translation at low temperatures (Schmid et al., 2009). The synthesis of cold acclimation proteins (Cap) also occurs during exposure to cold stress (Barria et al., 2013). Acid stress triggers mechanisms responsible for homeostasis maintaining, such as acid tolerance response (ATR), Glutamate decarbosylase activity (GAD), putative arginine deiminase (ADI) and F_1_F_0_-ATPase (Cotter et al., 2005) (Table 1; Figure 2). ATR protects the cell after short-term exposure to mild acids (Koutsoumanis et al., 2003). The GAD system enables survival in foods with low pH. GAD converts extracellular glutamate to gamma-aminobutyric acid (GABA), resulting in an increase in pHi (Werbrouck et al., 2009). Subsequently, GABA is exchanged for glutamate in the cell membrane via the GadT2 antiporter, contributing to the environment alkalization and pH homeostasis restoring (Ryan et al., 2009). In contrast, the ADI system is activated in response to extreme acid stress (low pH) (Soares and Knuckley, 2016). ADI converts, imported from the external environment, arginine to ornithine, CO_2_, ammonia and ATP. Ammonia, formed as a by-product, reacts with intracellular protons to produce NH^4+^, thereby increasing the pH of the cytoplasm and protecting the cell from an acidic environment (Soares and Knuckley, 2016). F_1_F_0_-ATPase generates a proton gradient, H^+^ efflux and restoration of homeostasis (Ryan et al., 2009) (Table 1; Figure 2).

The gastrointestinal stress-induced adaptive tolerance response to acid and osmotic stress can protect the pathogen from similar stresses in the gastrointestinal tract (GIT) and thus directly support its virulence potential. Moreover, in the GIT,*L. monocytogenes* switches from avirulent to a virulent state via reprogramming gene expression from stress survival-associated genes to virulence genes. The crosstalk between stress adaptation and pathogenicity is controlled by two overlapping and interrelated transcriptional networks regulated by sigma B factor and positive regulatory factor A (PrfA)) (Sibanda and Buys, 2022). The alternative sigma B factor - σ^B^, controls more than 300 stress response and virulence genes (Kazmierczak et al., 2003; Lungu et al., 2009). Researchers have shown that σ^B^ is involved osmotic stress, cold and heat stress, or oxidative stress response (Becker et al., 1998; Ferreira et al., 2001; Moorhead and Dykes, 2004). σ^B^ also contributes to the transcriptional activation of the *prfA* gene, encoding PrfA, the central regulator of *L. monocytogenes* virulence gene expression (Nadon et al., 2002). Recently, scientists have discovered new genes conditioning adaptation to unfavorable conditions, e.g., SSI-1 (Ryan et al., 2010) and SSI-2 (Harter et al., 2017). Importantly, hitherto unidentified genes were detected among the *L. monocytogenes* strains responsible for the outbreaks, e.g., LG-1 in the *L. monocytogenes* 08–5578 strain (the Canadian deli meat listeriosis outbreak in 2008) (Gilmour et al., 2010) and *bcrABC* gene in *L. monocytogenes* strain H7550 (the multistate outbreak in 1998–1999) (Elhanafi et al., 2010).

Despite extensive knowledge about the *L. monocytogenes* genome, the role of many proteins remains unexplained. An important aspect is also expanding knowledge on genomic and pathogenicity islands. A complete understanding of the virulent potential of *L. monocytogenes* strains requires whole genome data analysis combined with phenotypic characterization (Stratakos et al., 2020). In our opinion, exposure to stressful conditions can affect changes in the phenotypic characteristics of *L. monocytogenes*, which affects their virulence.

## Genomic and pathogenicity islands of *Listeria monocytogenes*

Genomic islands (GEIs) are gene clusters in the bacterial genome, most likely acquired through horizontal gene transfer (Gal-Mor and Finlay, 2006). Genomic islands contain genes that code for traits favorable under certain environmental conditions. GEIs are characterized by a large size (>10 kbp) and a different content of G + C (compared to the rest of the chromosome) (Dobrindt et al., 2004). GEIs can differ in the composition and sequence of genes even within one species (Hallstrom and McCormick, 2014). GEIs can evolve from mobile genetic elements, such as bacteriophages or plasmids, which can be transferred between unrelated microorganisms. A typical feature of many islands is the presence of a functional integrase gene, which allows for the insertion and removal of such elements (Dobrindt et al., 2004). This plasticity of GEIs enables pathogens adaptation to different environments (Dobrindt et al., 2004).

Pathogenicity islands (PAIs) are GEIs encoding virulence factors of pathogenic bacteria (Dobrindt et al., 2004; Hallstrom and McCormick, 2014). Non-pathogenic strains do not contain PAIs (Gal-Mor and Finlay, 2006). PAIs are a subclass of genomic islets obtained by horizontal transmission (Hallstrom and McCormick, 2014). PAIs can constitute large part of the chromosome (from 10 kbp to over 100 kbp) (Gal-Mor and Finlay, 2006). Expression of PAI genes, like other virulence genes, occurs in response to environmental cues (Gal-Mor and Finlay, 2006). Some strains also contain smaller pieces of DNA (1–10 kbp), termed “pathogenicity islets.” Expression of genes of PAIs is regulated by the transcription factors located on the island or externally (off the island) (Hallstrom and McCormick, 2014).

Infectious diseases, including listeriosis, remain a significant cause of mortality worldwide. The problem has been exacerbated recently by the increasing resistance of bacteria to antibiotics. Identifying the virulence factors used by these bacterial pathogens and understanding their evolution are relevant for both basic science and current medical challenges. The search and identification of various PAIs is essential from the medical point of view. Genes located on PAI can serve as markers in the molecular diagnostics of bacterial pathogens, assessment of their pathogenic potential, and even their antibiotic resistance pattern (Gal-Mor and Finlay, 2006).

The genome of *L. monocytogenes* is approximately 3 Mb, encoding approximately 2,910 core genes (den Bakker et al., 2010). *L. monocytogenes* virulence genes are organized within genomic and pathogenicity islands. Vázquez-Boland et al. (2001b) named the first *Listeria* spp. PAI LIPI-1, using a unified nomenclature to designate all large, genetically heterogeneous PAIs identified in *Listeria* spp. LIPI-1 plays a key role in the pathogenesis of *L. monocytogenes* due to the presence of genes required for the intracellular cycle (Portnoy et al., 1992; Vázquez-Boland et al., 2001b). Currently, scientists have identified in various strains of *L. monocytogenes* LIPI-1, LIPI-2 fragment (containing genes: *smcL*, *i-inIF* and *i-inIE*) (Yin et al., 2019), LIPI-3 (Cotter et al., 2008), LIPI-4 (Maury et al., 2016), and also genomic islands: LGI-1 (Gilmour et al., 2010), LGI-2 (Kuenne et al., 2013; Lee et al., 2013), LGI-3 (Palma et al., 2020) and SSI-1 (Ryan et al., 2009) and SSI-2 (Harter et al., 2017), which are discussed later in this paper.

Despite the broad knowledge of virulence and resistance determinants in*L. monocytogenes,* the functioning of many genes remains unexplained. More, scientists have been still reporting on new pathogenicity islands. We believe that understanding these genetic determinants is relevant for public health protection by limiting listeriosis outbreaks or reducing the rate of antibiotic resistance acquisition.

## Patgogenicity islands

### *Listeria* Pathogenicity Island 1 (LIPI-1)

**FIGURE 3:**
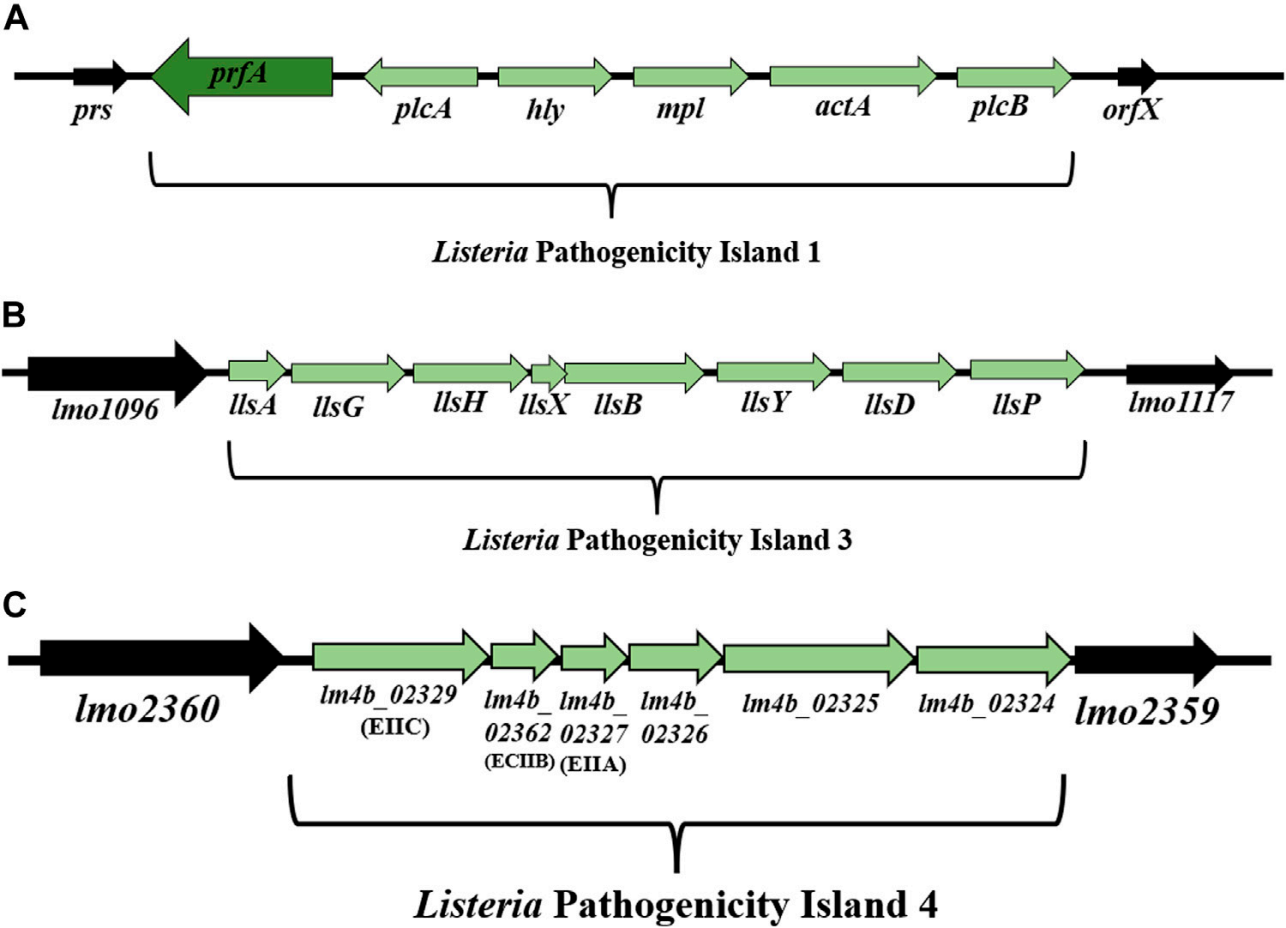
**(A)** Organization of *Listeria* Pathogenicity Island 1 (LIPI-1) (according to: Vázquez-Boland et al., 2001a; Vázquez-Boland et al., 2001b). **(B)** Organization of LIPI-3 among *L. monocytogenes* strains (according to: Cotter et al., 2008). **(C)** Organization of LIPI-4 among *L. monocytogenes* strains (according to: Maury et al., 2016). Direction of transcription is indicated by the respective arrows.

*Listeria* Pathogenicity Island 1 (LIPI-1) contains virulence genes involved in the intracellular infection cycle of *L. monocytogenes* (Vázquez-Boland et al., 2001a; Vázquez-Boland et al., 2001b; Osman et al., 2020). This 9 kbp gene cluster is located between *prs* and *orfX* and consists of six genes: *prfA*, *plcA*, *hly*, *mpl*, *actA*, and *plcB* (Dussurget, 2008; Hadjilouka et al., 2018) (Figure 3A). Down-stream of the *hly* gene is the *mpl-actA-plcB* operon of 5.7 kbp transcribed in the same orientation (Vázquez-Boland et al., 1992). In turn, the genes upstream of the *hly* gene, organized in the *plcA-prfA* operon, are transcribed in the opposite orientation (bicistronic or monocistronic) (Menguad et al., 1991; Freitag et al., 1993) (Figure 3A). The most intensive expression occurs at the physiological temperature of mammals (37°C), while the temperature drop to 30°C contributes to gene silencing (Mandin et al., 2005). LIPI-1 is regulated by the transcription factor PrfA (Cossart, 2011).

**FIGURE 4:**
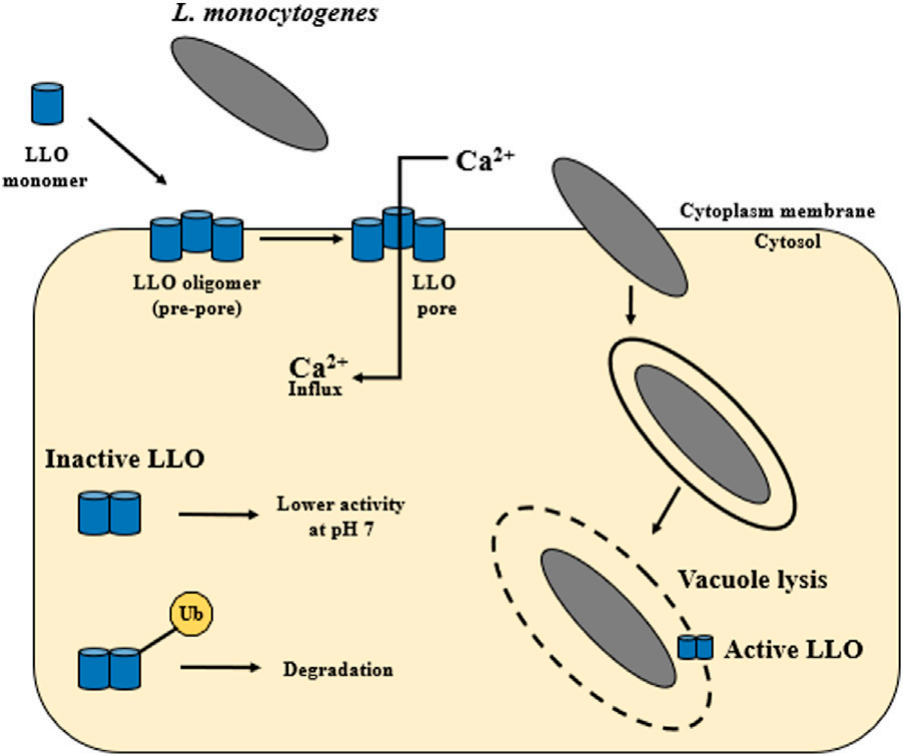
The activity of listeriolysin O. LLO is secreted as a water-soluble monomer that binds to cholesterol in host membranes. Then it oligomerizes into large complex—a toxin is formed, which can generate pores. The main function of LLO is the release of *L. monocytogenes* from the vacuole into the host cytoplasm. Additionally, the activity of LLO is pH dependent. Listeriolysin O has the so-called pH sensor. The highest activity of LLO is observed at the acidic pH of the vacuole (pH 5.5), while at the neutral pH of the host cytoplasm this activity decreases. Cytosolically synthesized LLO is first ubiquitylated and then LLO monomers are degraded by the proteasome. LLO participates in membrane internalization through membrane binding (cholesterol-rich) and oligomerization (pre-pore complex). This complex passes into transmembrane pores that allow the influx of extracellular Ca^2+^ (according to: Vadia et al., 2011; Arnett et al., 2014; Osborne and Brumell, 2017; Chen et al., 2018; Lam et al., 2018).

The *hly* gene (1,590 bp) encodes the pore-forming toxin listeriolysin O (LLO). LLO (58 kDa) was the first identified virulence factor of *L. monocytogenes* (Harvey and Faber, 1941; Gaillard et al., 1986; Geoffroy et al., 1987; Cossart et al., 1989). The main function of the LLO is to participate in the lysis of the phagocytic vacuole and the release of *L. monocytogenes* into the host cytoplasm (Arnett et al., 2014) (Figure 1). During spread to neighboring cells, *L. monocytogenes* is enclosed in a secondary vacuole (Figure 1) and then released by LLO to the host cytoplasm (Osborne and Brumell, 2017). Lam et al. (2018) have documented that LLO is critical for the human hepatocytes’ internalization (Figure 4). LLO participates in membrane binding (cholesterol-rich) and oligomerization (pre-pore complex). This complex passes into transmembrane pores that allow the influx of extracellular Ca^2+^. The increase of Ca^2+^ in the cytoplasm results in the translocation and activation of the conventional protein kinase C (cPKC). Activated cPKC signals induce Arp2/3-mediated remodeling of F-actin in the plasma membrane, leading to *L. monocytogenes* entry into the cell. The influx of extracellular Ca^2+^ also activates the membrane resealing pathway (Lam et al., 2018). LLO also forms small pores in the host cell membranes during other stages of the invasion cycle (Osborne and Brumell, 2017). LLO may play role in the cytoplasm of host cells. Pillich et al. (2012) have shown that the presence of LLO led to the induction of unfolded protein response (UPR). In contrast, damage to the endoplasmic reticulum (ER) (the site of intracellular calcium storage) caused by LLO is a source of calcium elevation during infection (Gekara et al., 2007).

Listeriolysin O belongs to the cholesterol-dependent cytolysin (CDC) family (Heuck et al., 2010). The proteins of the CDCs family have four regions. One of them (C-terminal domain) is involved in targeting the action of the toxin on the cytoplasmic membrane, and the other three are responsible for the oligomerization of LLO (Pizarro-Cerdá and Cossart, 2006). Köster et al. (2014) revealed the LLO crystal structure in 2014. According to the crystal structure, the LLO consists of four distinct domains (D1—D4), which play a different role in the functioning of the LLO (Köster et al., 2014). The D1 domain possesses a structural motif in the C-terminal region, crucial for membrane binding and the cytotoxic activity of the LLO (Faezi-Ghasemi and Kazemi, 2015; Materake and Okoh, 2020). In turn, the D2 domain is the sequence that joins D1 to D4 via a glycine linker (Rosado et al., 2008; Köster et al., 2014). The D3 domain consists of a five-stranded anti-parallel β sheet surrounded by six helices (Köster et al., 2014). A significant feature of D3 is the presence of three residues (D208, E247 and D320), i.e., a pH sensor (Dubail et al., 2001; Schuerch et al., 2005).

The LLO protein is synthesized as a precursor with the SS signal sequence at its N-terminus. Upon cleavage of the SS sequence, the mature protein functions as a monomer (Kayal and Charbit, 2006). In the first step, LLO is secreted as a water-soluble monomer that binds to cholesterol in the host’s membranes. Next, LLO oligomerizes into large complex (from 30 to 50 subunits). This stage results in a transmembrane toxin formation, generating pores with a diameter of about 50 nm (Dunstone and Tweten, 2012; Arnett et al., 2014; Chen et al., 2018) (Figure 4). Additionally, LLO activity is pH-dependent. Listeriolysin O has a so-called pH sensor (a triad of acidic amino acid residues in D3). The highest activity of LLO is observed at the acidic pH of the vacuole (pH 5.5), while at the neutral pH of the host cytoplasm this activity decreases (Schuerch et al., 2005; Bavdek et al., 2012). It ensures a quick inactivation of the toxin after *L. monocytogenes* escape into the cytoplasm. LLO requires a 30–40 mol% Chol threshold in the lipid membrane for effective binding and pore formation (Bavdek et al., 2007). Decatur and Portnoy (2000) have identified a relevant region within listeriolysin O, the so-called PEST-like region (P: proline, E: glutamine, S: serine, T: teronine). The PEST sequence is not required for the hemolytic activity but is a key element during the phagosomal escape of *L. monocytogenes* (Decatur and Portnoy, 2000). Due to six prolines, the PEST sequence shows the so-called Type II polyproline (PPII) system. The sequence plays a regulatory role in the host cytoplasm, inhibiting or preventing LLO oligomerization and pore formation (Kӧster et al., 2014). The vacuole rupture and access to the host cytoplasm occurs 15–30 min after infection of epithelial cells and macrophages (Quereda et al., 2018). LLO can also induce cytolysis in infected host cells even at a low concentration of 5 ng/mL (Jacobs et al., 1998).

The mechanism of LLO-mediated apoptosis induction on activated T cells involves two processes: one through caspase-3 and caspase-6 activation (Carrero et al., 2008). Caspase activation depends on the expression of granzymes (Carrero et al., 2008). Conversely, the second mechanism is LLO-dependent but caspase-independent, inducing phosphatidylserine exposure and loss of plasma membrane potential (Carrero et al., 2008). Listeriolysin O stimulates the host’s immune system, influences the production of pro-inflammatory mediators (NO), cytokines, and activates the NF - κB pathway and the formation of antibodies. LLO can additionally induce apoptotic pathways, stimulate MAP kinases (mitogen-activated protein kinases) and increase the expression of adhesion molecules (Camejo et al., 2011).

*L. monocytogenes* mutants that do not synthesize listeriolysin O remain trapped inside the vacuole and are five orders of magnitude less virulent than wild-type rods (Lam et al., 2018). The incidence of non-hemolytic *L. monocytogenes* is approximately 0.1% (Maury et al., 2017). Non-hemolytic *L. monocytogenes* strains most commonly occur among isolates from the food processing environment, but some clinical isolates have been reported to exhibit reduced hemolysis (Maury et al., 2017; Kawacka et al., 2022). Henry et al. (2006) have demonstrated that LLO deletion mutants escaped from the vacuole before being internalized in non-phagocytic human cell lines such as HeLa, HepG2, Henle 407, HEp-2, HCT116, HEK-293, and dendritic cells, where PlcB plays a major role in vacuole fracture.

LLO is one of the main toxins of *L. monocytogenes*, which determines virulence. However, researchers have attempted to use the toxin as a vaccine. In experiments carried out in animal models, researchers assessed LLO as an adjuvant in protective vaccinations against allergies, cancer and pathogens. The LLO was also used in the experimental treatment of tumor models such as follicular lymphoma and head and neck cancers (Xiong et al., 1998; Alberti-Segui et al., 2007; Hernández-Flores and Vivanco-Cid, 2015). The above studies offer the possibility of using this pathogen. Therefore, research on *L. monocytogenes* should also include the potential use of the toxins produced.

*L. monocytogenes* synthesizes two phospholipases C, specific for phosphatidylinositol, phospholipase A (PlcA, encoded by *plcA*, 954 bp) and broad-spectrum phospholipase B (PlcB, encoded by *plcB*, 870 bp). Both *plcA* and *plcB* genes are regulated by the transcription activator PrfA. These phospholipases hydrolyze phospholipids and then damage the host’s cytoplasmic membrane (Vázquez-Boland et al., 2001a; Vázquez-Boland et al., 2001b).

PlcB is a zinc-dependent metalloenzyme secreted as an inactive 264 amino acid proenzyme (to prevent degradation of the phospholipids contained in the bacterial membrane). PlcB (29–30 kDa) is activated by proteolytic cleavage in the extracellular environment. There is also an Mpl-independent activation path. This pathway depends on the level of acidification of the vacuole environment (Vázquez-Boland et al., 2001a). PlcB has a wide range of optimum pH - from 5.5 to 8.0. PlcB hydrolyzes phosphatidylcholine, phosphatidylethanolamine, phosphatidylserine, and sphingomyelin. It shows weak phosphatidylinositol hydrolysis activity and weak calcium-independent hemolytic activity at 37°C (Vázquez-Boland et al., 2001a). PlcB plays a relevant role in vacuole escape and spread from cell to cell (Alberti-Segui et al., 2007; Quereda et al., 2018).

PlcA is a phospholipase that cleaves the signal between alanine (29) and tyrosine (30). This enzyme is specific for phosphatidylinositol (PI) but also slightly hydrolyzes eukaryotic PI glycosyl (GPI)—a membrane protein with an optimal pH range of 5.5–7.0 (Goldfine and Knob, 1992). PlcA helps in the escape from the primary phagosomes and the secondary binary vacuolar membrane (Alberti-Segui et al., 2007).

Surface protein ActA (Actin assembly-inducing protein) ensures intra- and intercellular movement of*L. monocytogenes* (Py et al., 2007). The ActA protein is responsible for the polymerization of actin filaments in 1 cell pole, creating a structure resembling the so-called “Comet tail” (Camejo et al., 2011). The resulting force allows the rods to move within the host’s cytoplasm, form a secondary phagosome, and to enter the neighboring cell (Py et al., 2007; Camejo et al., 2011). ActA interacts with the Arp2/3 complex proteins, VASP (vasodilator-stimulated phospho-protein), profilin and cophilin (Pizarro-Cerdá and Cossart, 2006). The central ActA domain (proline-rich region) interacts with proteins of the Ena/VASP complex that modulate the speed and direction of rod movement. VASP recruits profilins (actin monomer-binding proteins), which enables actin polymerization. The protein reduces the frequency of branching of actin fibers, which promotes the formation of long and parallel filaments (Rafelska and Therio, 2006; Camejo et al., 2011). Also ERM family proteins (ezrin, radixin and moesin) contribute to efficient spread of *L. monocytogenes* to neighboring cell. ERM proteins connect the actin tail with the cytoplasmic membrane, forming and stabilizingsecondary sections in the membrane (Pust et al., 2005). The ActA protein may have other functions, e.g., binding heparan sulfate on the cell surface, which allows attachmemt and entering host cells cultured *in vitro* (Alvarez-Dominguez et al., 1997; Suárez et al., 2001). In addition, the ActA protein enables the spread of *L. monocytogenes* within the placenta (Bakardjiev et al., 2005; Le Monnier et al., 2007). There is also a model in which ActA is involved in crossing the blood-brain barrier, at least in part, through the so-called “Trojan horse” mechanism, which has not been formally confirmed yet (Drevets et al., 2001; Join-Lambert et al., 2005). ActA expression depends on the growth medium composition and temperature (Travier and Lecuit, 2014). Moreover, full activation of *actA* under varying environmental conditions, such as low temperature, requires both PrfA and σ^B^ (Tiensuu et al., 2013). Different versions of the *actA* gene are present in hypervirulent strains of *L. monocytogenes*. Hence, the effect of mutations in the *actA* gene on virulence and pathogenicity in humans is not fully understood (Lake et al., 2021).

Metalloprotease (Mpl) has the HEXXH motif characteristic of this family members. Metal-loprotease is synthesized in the form of a proenzyme (Bitar et al., 2008) and activates phospholipase B (PC - PLC) (Camejo et al., 2011). Mutants with a transposon insertion in *mpl* gene exhibited reduced virulence and lecithinase production (Menguad et al., 1991; Raveneau et al., 1992). Poyart et al. (1993) highlighted that a zinc-dependent metalloprotease is involved in the virulence of *L. monocytogenes* through its action on PC-PLC. A study by Alvarez and Agaisse (2016) showed that Mpl regulates ActA levels on the bacterial side in protrusions. The scientists have suggested that Mpl maintains ActA polymerization in protrusions which contributes to efficient actin polymerization (Alvarez and Agaisse, 2016). The *mpl*, *plcB* and *actA*genes are organized in one operon (Hain et al., 2006).

**FIGURE 5:**
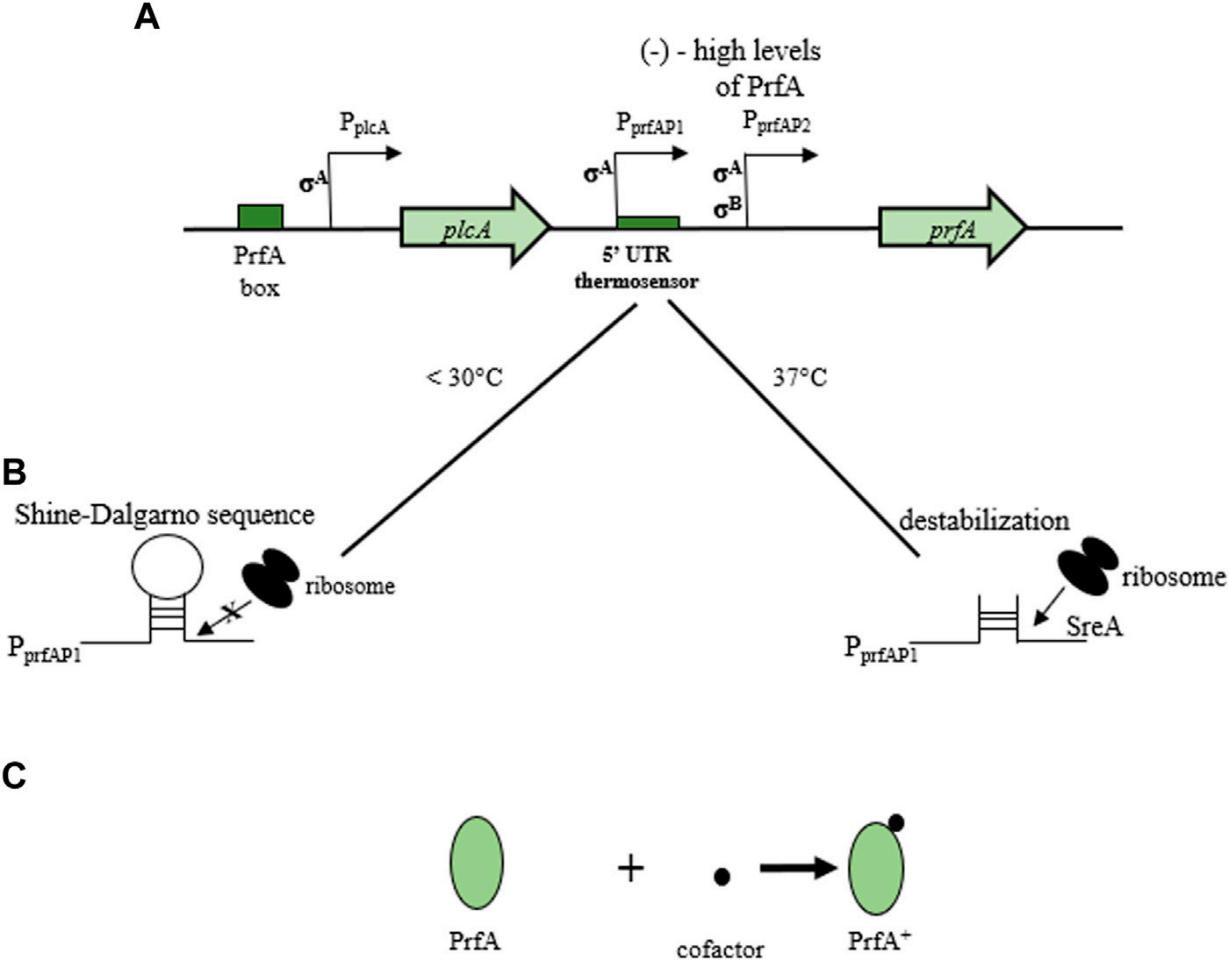
Regulation of *prfA* expression and PrfA protein activity. **(A)** transcriptional control of *prfA* (three promoters of *prfA* gene). P_plcA_ positively directs *prfA* expression by binding to the “PrfA box”. P_prfAP1_ and P_prfAP2_ control monocistronic reactions. The P_prfAP1_ promoter has a 5′UTR untranslated region that acts as a temperature sensor); **(B)** post-transcriptional control of *prfA* (at 30°C, a stable spatial structure, referred to as a “hairpin” is formed in the region containing the Shine-Dalgarno sequence. It unifies the attachment of the ribosome and the synthesis of the polypeptide. At 37°C, the “hairpin” structure is destabilized, allowing attachment of the ribosome and synthesis of the PrfA protein); **(C)** post-translational control of PrfA (attachment of a cofactor molecule (glutathione) to the PrfA protein) (according to: Loh et al., 2009; Xayarath and Freitag, 2012; Lemon et al., 2010; Johansson and Freitag, 2019).

The *prfA* gene can exist in two functional states, i.e., weakly or highly active. Its activity depends on temperature, presence of carbon, and easily metabolized sugars such as cellobiose (McGann et al., 2007). The PrfA protein (27 kDa) is a member of the CRP (cyclic AMP receptor protein)/FNR (fumarate and nitrate reduction regulator) family and is composed of 233 amino acids (Arnett et al., 2014). Polypeptide expression and activity regulation includes transcription, post-transcriptional and post-translational mechanisms (Port and Freitag, 2007). The *prfA* gene recognizes a 14 bp palindromic sequence termed “PrfA box” situated typically 40 nucleotides upstream of the target transcription start site. In the post-transcriptional mechanism, the level of PrfA activity depends on environmental factors (the presence of fermentable carbohydrates) and the physiological state of the rods (Ollinger et al., 2009). The fully active state of PrfA in the post-translational mechanism ensures the binding of a small cofactor molecule (Mueller and Freitag, 2005). The *prfA* gene has three promoters. P_plcA_ positively directs *prfA* expression by binding to the so-called “PrfA box” (bicistronic expression). In contrast, P_prfAP1_ and P_prfAP2_ control monocistronic reactions. The P_prfAP1_ promoter has a 5′UTR untranslated region that acts as a temperature sensor (Figure 5A). Transcript translation is only effective at 37°C. At the temperature of 30°C, in the region containing the Shine-Dalgarno sequence, a stable spatial structure of the mRNA is formed, preventing the attachment of the ribosome and the polypeptide synthesis. At the physiological temperature of the host (37°C), destabilization of the structure occurs, enabling the PrfA synthesis (Figure 5B). The expression of the P_plcA_ and P_prfAP2_ promoters is independent of temperature (Lemon et al., 2010). The P_prfAP1_ promoter is recognized by the factor σ^A^, while P_prfAP2_ can be recognized by σ^A^ and σ^B^ factors (Lemon et al., 2010). Full PrfA activation requires cofactor, i.e., glutathione binding allosterically to the protein (Johannson and Freitag, 2019) (Figure 5C). According to Loh et al. (2009), expression of the *prfA* gene is almost 16-fold higher at 37°C compared to 30°C. PrfA regulates expression of LIPI-1 genes (Cossart, 2011).

A recent analysis by Prokop et al. (2017) has showed that the *orfX* gene (*lmo0206*) also plays a significant role in the virulence of *L. monocytogenes*. Researchers showed that OrfX is a small se-creted protein positively regulated by PrfA. The primary role of OrfX is to suppress the oxidative reaction of infected macrophages, which contributes to the intracellular survival of the bacteria. OrfX targets the nucleus and lowers the regulatory protein RybP levels (Prokop et al., 2017).

Genes located on LIPI-1, i.e., *prfA* and *hly*, are commonly used for *L. monocytogenes* detection, especially in food products (using PCR reaction) (Amagliani et al., 2004; Wang et al., 2004; Jofre et al., 2005; Germini et al., 2009). Ward et al. (2004) reported that phylograms from each of the genes present in LIPI-1 can differentiate the strains studied according to their origin. Also, Hadjilouka et al. (2018) have revealed that *plcA*, *plcB*, *mpl*, *actA* and intergenic regions *plcA-prfA* and *plcA-hly* are useful for serotypes differentiation. In turn, Poimenidou et al. (2018) have reported that LIPI-1 virulence genes follow different evolutionary paths. Evolutionary changes depend on the strain origin and serotype, as well as the epidemiological dominance of some subgroups. Additionally, research has shown that the most conserved genes are *prfA* and *hly*, and the *actA* gene is the most diverse (Poimenidou et al., 2018).

LIPI-1 plays essential role in the virulence of *L. monocytogenes*. Due to LIPI-1 genetic diversity it could be valuable to investigate the role of point mutations in the pathogenicity and stress adaptation of *L. monocytogenes*.

### *Listeria* Pathogenicity Island 2 (LIPI-2)

The LIPI-2 region is specific for strains belonging to the species *Listeria ivanovii*. On LIPI-2 (22 kbp) the following genes are present: *i-inlB2*, *i-inlL*, *i-inlK*, *i-inlB1*, *i-inlJ*, *i-inlI*, *i-inlH*, *i-inlG*, *smcL*, *i-inlF*, *i-inlE*, *surF3*, mainly coding internalins (Sergeant et al., 1991; Guillet et al., 2010). LIPI-2 in *L. ivanovii* is located between *lmo1240* and *lmo1422* (Vázquez-Boland et al., 2001a). However, Yin et al. (2019) have documented the LIPI-2 locus (presence of LIPI-1 and absence of LIPI-3 and LIPI-4) in *L. monocytogenes* isolates (HSL-II, serovar 4h) responsible for the listeriosis outbreak in China. The identified LIPI-2 fragment contained the *smcL*, *i-inIF* and *i-inIE*, genes encoding sphingomyelinase and internalin, respectively. This fragment *L. monocytogenes* likely acquired through the exogenous DNA acquisition from *L. ivanovii* (Yin et al., 2019). The *i-inlEF* locus is characteristic of *L. ivanovii*. Both the *i-inlE* and *i-inlF* genes are under the control of PrfA. The *i-inlF* and *i-inlE* genes are arranged in tandem, which suggests they generation by gene duplication (Engelbrecht et al., 1998). In turn, the *smcL* gene (1,008 bp) is PrfA-independent, and sphingomyelinase C (335 amino acids) is responsible for the different hemolytic properties of *L. ivanovii* (bizonal hemolysis and CAMP-like reaction with *Rhodococcus equi*). González-Zorn et al. (2000) have shown that the 5′end of the *smcL* gene was contiguous with the *i-inlFE* locus. The role of the LIPI-2 locus among *L. monocytogenes* HSL-II has not been elucidated yet (Disson et al., 2021). According to Yin et al. (2019), the LIPI-2 fragment presence may result from the kinship and coexistence of *L. ivanovii* and *L. monocytogenes* in the same environment. Additionally, HSL-II strains possessed many other virulence factors associated with cases of listeriosis in humans (Yin et al., 2019). The accquisition of a new PAI can significantly affect the phenotype or lifestyle of *L. monocytogenes*. Therefore, there is a need for further studies on the identification and the role of LIPI-2 in *L. monocytogenes*.

### *Listeria* Pathogenicity Island 3 (LIPI-3)

Cotter et al. (2008) have demonstrated the presence of *Listeria* Pathogenicity Island 3 (LIPI-3) among *L. monocytogenes* strains line I F2365 (SL1/CC1) and H7858 (SL6/CC6) (Cotter et al., 2008). LIPI-3 consists of eight genes: *llsA*, *llsG*, *llsH*, *llsX*, *llsB*, *llsY*, *llsD*, *llsP* (Figure 3B) (Cotter et al., 2008; Disson et al., 2021).

The operon LLS encodes listeriolysin S (LLS, a thiazole/oxazole–modified microcin (TOMM)), a post-translational modified peptide that exhibits properties of both bacteriocin and hemolytic cytotoxic factor (Cotter et al., 2008; Quereda et al., 2016; Meza-Torres et al., 2021). The LLS operon consists of a structural gene encoding a peptide (*llsA*), three genes that form the synthetase complex necessary for LLS maturation (*llsB*, *llsY*, *llsD*), an ABC transporter (*llsG*, *llsH*), a putative protease (*llsP*), and a gene of unknown function (*llsX*) (Clayton et al., 2011; Molloy et al., 2011; Quereda et al., 2016; Quereda et al., 2017; Lee, 2020). As a bacteriocin, LLS restricts the growth of other related Gram-positive bacteria such as *Lactococcus lactis*, *Lactobacillus plantarum*, *Staphylococcus aureus* and even *L. monocytogenes* line II (EGD and 10403S) that lack the LLS operon (Cotter et al., 2008; Mohammadzadeh et al., 2019). LLS causes only weak hemolysis of red blood cells *in vitro*, and is not cytotoxic to eukaryotic cells (Cotter et al., 2008). The LLS probably contains the Ala-Gly motif (amino acid 26), and the C-terminal core region with an extreme predominance of Cys, Ser and Thr residues allowing post-translational modifications resulting in a characteristic heterocyclic compound (Clayton et al., 2011). Meza-Torres et al. (2021) have shown that LLS remains bound to the bacterial cell membrane and cytoplasm and is not secreted into the extracellular space of the bacteria. LLS requires direct contact between LLS-producing bacteria and target bacteria in order to exhibit bactericidal activity and thus behaves like a contact-dependent bacteriocin. Contact exposure to LLS leads to permeabilization/depolarization of the target bacterial cell membrane and release of adenosine triphosphate (ATP) (Meza-Torres et al., 2021). Cotter et al. (2008) have noted that *llsA* promoter expression was negligible *in vitro* and only induced upon exposure to hydrogen peroxide. In turn, Quereda et al. (2016) have found slight and enhanced *llsA* expression under classical *in vitro* laboratory conditions and in infected mice, respectively. The discrepancy between the two studies requires further experimentation. The LLS cluster is present only in the subset of line I strains, responsible for the majority of human listeriosis outbreaks, and absent in line II and III strains of *L. monocytogenes* (Cotter et al., 2008).

The *llsX* gene encodes a potential membrane signal peptide of unknown function but very specific for *L. monocytogenes*. Researchers have already identified the *llsX* gene in strains with different origins and genetic profiles (Chen et al., 2018; Kim et al., 2018). Tavares et al. (2020) have shown that the *L. monocytogenes* strains belonging to line II did not possess LIPI-3. Researchers have also demonstrated *llsX* expression under acidic stress after 6-h incubation for one of the three tested strains of serogroup 4b (Tavares et al., 2020). In addition, Vilchis-Rangel et al. (2019) showed a strong relationship between *llsX* and the invasiveness of *L. monocytogenes*. Several studies (Clayton et al., 2011; Chen et al., 2019; Wang et al., 2019) have used the *llsX* gene as a marker of LIPI-3, provided that this gene is well conserved in various clonal complexes of *L. monocytogenes*, and even in atypical hemolytic *Listeria innocua*.

The *llsB* gene putatively plays an essential role in the systemic infection phase (Chen C. et al., 2018). The functions of the remaining genes still require analysis.

The*llsB* gene likely plays an essential role in the systemic infection phase (Chen C. et al., 2018). The functions of the remaining genes still require analysis.

Since the discovery of LIPI-3, LIPI-3 has been documented among many*L. monocytogenes* isolates worldwide (Kim et al., 2018; Camargo et al., 2019; Zhang et al., 2019; Chen Y. et al., 2020a; Wieczorek et al., 2020; Zhang et al., 2020), belonging to different lineages. Further research should focus on the role of all genes located on LIPI-3, especially of unknown function (*llsX*).

### *Listeria* Pathogenicity Island 4 (LIPI-4)

The presence of *Listeria* Pathogenicity Island 4 (LIPI-4) has been demonstrated among clinical strains isolated from infections of the central nervous system and placenta (Maury et al., 2016), as well as among *L. monocytogenes* SL87 (CC87) strains widespread in Asia (Wang et al., 2019). LIPI-4 (6 kbp) is located between the genes *lmo2360* and *lmo2359* (Maury et al., 2016; Disson et al., 2021). LIPI-4 encodes the cellobiose family phosphotransferase system that determines the tropism of *L. monocytogenes* to the central nervous system (CNS) and placental cells (Maury et al., 2016). Genes located within LIPI-4 include: *lm4b_02324* (maltose-6′-P-glucosidase), *lm4b_02325* (transcriptional antiterminator), *lm4b_02326* (uncharacterized protein associated to PTS systems), *lm4b_02327* (membrane permease EIIA), *lm4b_02328* (membrane permease EIIB), *lm4b_02329* (membrane permease EIIC) (Figure 3C) (Maury et al., 2016). Lake et al. (2021) have found LIPI-4 among all CC4 (serogroup 4b-4d-4e) and CC87 strains (serogroup 1/2b-3b-7). In turn, Shen et al. (2022) have identified LIPI-4 in CC4 and CC388. The presence of LIPI-4 indicates the hypervirulent character of *L. monocytogenes* strains (Maury et al., 2016; Hurley et al., 2019; Raschle et al., 2021). Identification of LIPI-4 among subsequent clones of *L. monocytogenes*, both clinical and environmental strains (Raschle et al., 2021), confirms the need to continue research on the function of the remaining proteins and monitoring of *L. monocytogenes* in the environment. Due to the involvement of genes located on LIPI-4 in the nervous system and placenta infections, screening of strains from all evolutionary lineages is advisable.

## Genomic islands

### *Listeria* Genomic Island-1 (LGI-1)

Genomic islands may contain genes that potentially influence higher adaptation to unfavorable environmental conditions. It, in turn, may increase the pathogenic potential of bacteria. Gilmour et al. (2010) has demonstrated for the first time a horizontally acquired LGI-1 (*Listeria* Genomic Island 1, coordinates 1836435-1886209 of 08-5578; coding sequences LM5578_1850 to LM5578_1903) in strains of *L. monocytogenes* serotype 1/2a isolated from the 2008 listeriosis outbreak in Canada. It was the deadliest outbreak in Canada, and the source of the pathogen was deli meat. LGI-1 isolates studied so far belonged to serotype 1/2a except for the one isolate of serotype 3a (Knabel et al., 2012). The island of LGI-1 (50 kb) encodes genes responsible for virulence, resistance to antimicrobial substances, and stress factors. The canonical genes predicted in LGI-1 include *virB4, virD4*, and *virB11*, which encode ATPases recruiting substrates into the cell, and *virB5* and *virB6*, or subunit genes that form the core of the membrane transfer complex. The presence of the *cpa* and *tad* genes indicates possible pilus-like outgrowth, whereas the *dnaG* gene presence suggests that the genetic island may be mobilized (Gilmour et al., 2010). Researchers have also identified the *emrE* gene encoding an efflux pump involved in the resistance to toxic cationic hydrophobic molecules such as quaternary ammonium compounds and tetracycline (Pornillos et al., 2005; Gilmour et al., 2010). A study by Kovacevic et al. (2016) has demonstrated that the minimal function of the LGI-1 island enhances *L. monocytogenes* tolerance to quaternary ammonium compounds (QAC) through *ermELm*. The data on LGI-1 presented so far indicate its great importance in the survival of *L. monocytogenes* within the food processing chain and during host invasion. To date, LGI-1 has not been identified among many strains in studies conducted around the world (Table 3).

**TABLE 3:** Summary of selected papers (published in 2018–2022) describing the frequency of genomic/pathogenicity islands among *L. monocytogenes* strains isolated from various sources.

Country	Year	Source	Isolates	Presence of pathogenic/genomic islands (n (%))	References
China (provinces: Zhejiang, Fujian, Hebei, Henan, Beijing, Xinjiang)	2002–2019	food (326); livestock (25); clinical (18)	369	LIPI-1—369 (100.00%)	Anwar et al. (2022)
				LIPI-3—36 (10.00%)	
				LIPI-4—33 (9.00%)	
43 cites in China	2012–2016	meat and meat products	362	LIPI-3—37 (10.22%)	Chen et al. (2019)
				LIPI-4—75 (20.72%)	
China (Shanghai)	2009–2019	food	155	LIPI-1—155 (100.00%)	Zhang et al. (2020)
				LIPI-3—12 (7.74%)	
				LIPI-4—21 (13.55%)	
China (collected at Shanghai port)	2018–2020	imported foods (pork, fish, sheep, chicken, beef)	81	LIPI-1—81 (100.00%)	Shen et al. (2022)
				LIPI-3—16 (19.75%)	
				LIPI-4—5 (6.17%)	
				SSI-1—46 (56.79%)	
				SSI-2—8 (9.88%)	
China	2012–2015	food	28	LIPI-1—28 (100.00%)	Yan et al. (2019)
				LIPI-3—2 (7.14%)	
				LIPI-4—0 (0.00%)	
				SSI-1—27 (96.43%)	
43 cites in China	2012–2016	fresh aquatic products	72	LIPI-1—72 (100.00%)	Chen M. et al. (2018)
				LIPI-3—8 (11.11%)	
				LIPI-4—16 (22.22%)	
China (Wuhan)	2019	retinal pork	64	LIPI-1—64 (100.00%)	Wang et al. (2021)
				LIPI-3—6 (9.38%)	
				LIPI-4—5 (7.81%)	
21 cites in China	2014–2016	ready-to-eat foods and pasteurized milk	48	LIPI-1—48 (100.00%)	Chen Y. et al. (2020a)
				LIPI-3—6 (12.5%)	
				LIPI-4—15 (31.25%)	
China (Beijing)	2014–2018	clinical	151	LIPI-1—150 (99.38%)	Zhang et al. (2021)
				LIPI-3—26 (17.22%)	
				LIPI-4—42 (27.81%)	
Japan	2006–2019	clinical	18	LIPI-1—18 (100.00%)	Baba et al. (2021)
				LIPI-3—8 (44.44%)	
				LIPI-4—1 (5.56%)	
United States (California, Maryland, Connecticut, and Georgia)	2010–2013	ready-to-eat food samples	100	LIPI-1—100 (100.00%)	Chen Y. et al. (2020b)
				LIPI-3—25 (25.00%)	
				LIPI-4—15 (15.00%)	
				SSI-1—58 (58.00%)	
				SSI-2—1 (1.00%)	
United States (North Dakota, South Dakota, Minnesota, Nebraska, and Michigan)	2015–2020	ruminant listeriosis cases	73	LIPI-1—73 (100.00%)	Cardenas-Alvarez et al. (2022)
				LIPI-3—23 (31.51%)	
				LIPI-4—10 (13.70%)	
				SSI-1—7 (9.59%)	
				SSI-2—6 (8.22%)	
				LGI-2—1 (1.40%)	
				LGI-3—1 (1.40%)	
United States	2002–2014	isolated recovered from bulk tank milk, milk filters, and milking equipment from dairies	121	LIPI-1—121 (100.00%)	Kim et al. (2018)
				LIPI-3—46 (38.02%)	
				LIPI-4—21 (17.36%)	
				SSI-1—54 (44.63%)	
				LGI-1—0 (0.00%)	
United States (New York)	2018–2019	wildlife	13	LIPI-1—13 (100.00%)	Chen et al. (2022)
				LIPI-3—4 (30.77%)	
				LIPI-4—3 (23.08%)	
				SSI-1—4 (30.77%)	
				SSI-2—0 (0.00%)	
United States (Central California Coast)	2011–2016	surface waters in agricultural region	1,248	LIPI-1—1,248 (100.00%)	Gorski et al. (2022)
				LIPI-3—913 (73.20%)	
				LIPI-4—785 (62.90%)	
				SSI-1—151 (12.00%)	
				SSI-2—0 (0.00%)	
				LGI-2—50 (4.00%)	
Brazil	1978–2013	food production environment, beef, clinical	35	LIPI-1—35 (100.00%)	Camargo et al. (2019)
				LIPI-3—15 (43.00%)	
				LIPI-4—2 (6.00%)	
				SSI-1—20 (57.00%)	
				SSI-2—3 (2.86%)	
				LGI-1—8 (22.86%)	
Chile	2008–2011	clinical (22); food and food-related environments	38	LIPI-1—38 (100.00%)	Toledo et al. (2018)
				LIPI-3—16 (42.11%)	
				SSI-1—14 (36.84%)	
				SSI-2—32 (5.26%)	
Mexico (Guadalajara)	No data	obtained from Hass avocados sold at retail markets	18	LIPI-1—7 (38.895)	Avila-Novoa et al. (2021)
Mexico	No data	food (19); clinical (1)	20	LIPI-1—19 (95.00%)	Vilchis-Rangel et al. (2019)
				LIPI-3—7 (35.0%)	
South Africa	2014–2019	red meat and poultry value chain	217	LIPI-1—16 (7.40%)	Matle et al. (2020)
				LIPI-3—47 (21.70%)	
				LIPI-4—4 (1.80%)	
Spain (Cantabria region)	2017–2019	isolated from Dairy Cattle Farms	45	LIPI-1—45 (100.00%)	Varsaki et al. (2022)
				LIPI-3—39 (86.67%)	
				LIPI-4—9 (20.00%)	
Central Italy	2020–2021	isolated in a meat producing plant	84	LIPI-1—84 (100.00%)	Guidi et al. (2022)
				LIPI-3—84 (100.00%)	
Irleand	2009–2014	three food processing environments	100	LIPI-1—100 (100.00%)	Hurley et al. (2019)
				LIPI-3—10 (10.00%)	
				LIPI-4—1 (1.00%)	
				SSI-1—51 (51.00%)	
				SSI-2—1 (1.00%)	
Netherlands	spring 2018	isolated during mushroom production and processing	44	LIPI-1—44 (100.00%)	Lake et al. (2021)
				LIPI-3—30 (68.18%)	
				LIPI-4—14 (31.82%)	
				SSI-1—17 (36.64%)	
				SSI-2—0 (0.00%)	
Switzerland	between January and May 2020	flowing surface waters	25	LIPI-1—25 (100.00%)	Raschle et al. (2021)
				LIPI-3—12 (48.00%)	
				LIPI-4—4 (16.00%)	
				SSI-1—3 (12.00%)	
Poland	2019	isolates from fish manufactures	28	LIPI-1—28 (100.00%)	Wieczorek et al. (2020)
				LIPI-3—1 (3.57%)	
				SSI-1—10 (35.71%)	
				SSI-2—14 (50.00%)	
Poland	2014–2017	food (33); food processing environment (15)	48	LIPI-1—48 (100.00%)	Kurpas et al. (2020)
				LIPI-3—15 (31.30%)	
				LIPI-4—0 (0.00%)	
Germany	2008–2016	food production plants	93	LIPI-1—93 (100.00%)	Roedel et al. (2019)
				LIPI-3—14 (15.05%)	
				LIPI-4—1 (1.08%)	
				SSI-1—40 (43.01%)	
				SSI-2—8 (8.60%)	
				LGI-1—0 (0.00%)	
				LGI-2—19 (20.43%)	
Australia	1998–2016	dairy, meat, vegetable, mixed food and environment	52	LIPI-1—52 (100.00%)	Gray et al. (2021)
				LIPI-3—5 (9.62%)	
				LIPI-4—0 (0.00%)	
				SSI-1—34 (65.38%)	
				SSI-2—5 (9.52%)	
				LGI-1—0 (0.00%)	
				LGI-2—8 (15.38%)	
				LGI-3—5 (9.62%) (no homolog of the *cadAC* gene)	
China, Canada, Switzerland, United States, Italy	2012–2021	clinical	60	LIPI-1—60 (100.00%)	Shi et al. (2021)
				LIPI-3—14 (23.33%)	
40 countries from six continents	1921–2018	from human hosts (1,453); animals (44); food (387); food processing environments (88); feed (11); natural environments (11); unknown sources (27)—only clonal complex 1 (Lm-CC1)	2021	LIPI-1—2021 (100.00%)	Moura et al. (2021)
				LIPI-3—2021 (100.00%)	
				LGI-2—277 (14.00%)	

LIPI, *Listeria* pathogenicity island; LGI, *Listeria* Genomic Island; SSI, Stress Survival Islet.

### *Listeria* Genomic Island-2 (LGI-2)

The genome of *L. monocytogenes* Scott A possesses a 35 kb chromosomal region called *Listeria* Genomic Island 2 (LGI-2) (Kuenne et al., 2013; Lee et al., 2013; Lee et al., 2017). LGI-2 contains a cassette of arsenic resistance genes (*arsR1D2R2A2B1B2*), two additional preceding genes (*arsD1A1*), cadmium resistance gene (*cadA4*)), and genes putatively implicated in DNA integration, conjugation, and pathogenicity (Brires et al., 2011; Lee et al., 2013; Parsons et al., 2017). LGI-2 has been identified mainly among *L. monocytogenes* serotype 4b strains, including hypervirulent clones of serotype 4b CC1 and CC2 (Kathariou et al., 2017; Lee et al., 2017; Gelbicova et al., 2021), and several stable strains belonging to CC14 and CC204 line II (Fox et al., 2016; Lee et al., 2017; Pasquali et al., 2018). Lee et al. (2017) have observed content plasticity of LGI-2. Researchers have identified LGI-2 at multiple genomic locations, frequently disrupting open frame reading and shown sequence content variation (Lee et al., 2017).

Researchers have detected notably diverse variant of this island, designated LGI2-1, in some strains of *L. monocytogenes* CC1 (Lee et al., 2017). LGI2-1 contained a unique gene encoding a putative cystathionine gamma-synthase immediately upstream of arsenic resistance genes (Lee et al., 2017; Haubert et al., 2019). The ability of strains possessing LGI2-1 to tolerate higher levels of cadmium seems to be mediated by *cadA5C5* (Lee et al., 2017). The authors have suggested that LGI-2 may serve as a model showing dynamic selection of arsenic-resistant subpopulations of *L. monocytogenes* after exposure to a strong environmental toxin such as arsenic (Lee et al., 2017).

There is limited information on the potential effect of heavy metal tolerance on the virulence and persistence of *L. monocytogenes*. However, understanding the ecology and evolution of LGI-2 among hypervirulent strains of *L. monocytogenes* capable of cadmium and arsenic detoxification would be significant.

### *Listeria* Genomic Island-3 (LGI-3)

Palma et al. (2020) have revealed *Listeria* Genomic Island-3 (LGI3) (31.5 kbp) in *L. monocytogenes* strains (isolated in France) as a highly conserved and specific region for CC101 strains (persistent, RTE seafood processing plants). LGI-3, located downstream of the *inIJ* gene (homologue of strain EGD-e *lmo1413*) (Palma et al., 2020), contains 29 predicted coding sequences. It integrates the chromosomal cadmium resistance determinant *cadA1C*, flanked by recombinase and Tn3 transposase, and genes putatively implicated in DNA integration, conjugation, translocation, and recombination. Palma et al. (2020) have suggested the need for a more detailed characterization of LGI-3 in order to know the virulent potential of *L. monocytogenes*. Persistent strains of *L. monocytogenes* are defined as isolates repeatedly isolated from the same source or ecological niche over a period of time (Unrath et al., 2021). Scientists have recently confirmed the presence of persistent strains in the food processing environment (Ferreira et al., 2014; Leong et al., 2014; Wiktorczyk-Kapischke et al., 2022). Undoubtedly, surviving strains are a significant global problem. Understanding the genetic aspects of the environmental persistence of *L. monocytogenes* strains would be highly relevant.

### Genomic island 7 (GI-7)

As already, Yin et al. (2019) have mentioned demonstrated the LIPI-2 fragment among the hybrid subline II strains of *L. monocytogenes* serovar 4h. The latest study by Jin et al. (2022) identified the *LMxysn_1693* gene, a component of genomic island-7 (GI-7) (containing 20 ORFs) in the 4h serovar of *L. monocytogenes* XYSN strain (high-virulent). *LMxysn_1693* gene (534 bp) synthetically interacts with genes involved in bile resistance and biofilm formation, thus contributing to resistance in the intestinal environment. More, *LMxysn_1693* can upregulate the transcriptional expression of PrfA. Therefore, the interaction between PrfA and LMxysn_1693 affects the ability of *L. monocytogenes* to form a biofilm (Jin et al., 2022). The GI-7 island likely contains two ABC transporters involved in extracellular and surface proteins transport (Jin et al., 2022). ABC transporters are relevant for the functioning of bacteria, especially during invasion (Locher, 2016), but also participate in biofilm formation (Benda et al., 2021), resistance to environmental stress conditions (Kang et al., 2015; Grubaugh et al., 2018; Jiang et al., 2019) and metal utilization (Kang et al., 2015; Grubaugh et al., 2018; Jiang et al., 2019). Most GI-7 genes encode hypothetical proteins of unknown function. Therefore, further studies on the role of GI-7 are required.

Genomic islands determine virulence and resistance determinants in *L. monocytogenes*. However, the composition and sequence of genes within GEIs can vary, making their identification a bit challenging. We believe that whole genome sequencing can be a helpful tool to search for GEIs among *L. monocytogenes*.

### Stress survival islets

#### Stress survival islet (SSI)-1

**FIGURE 6:**
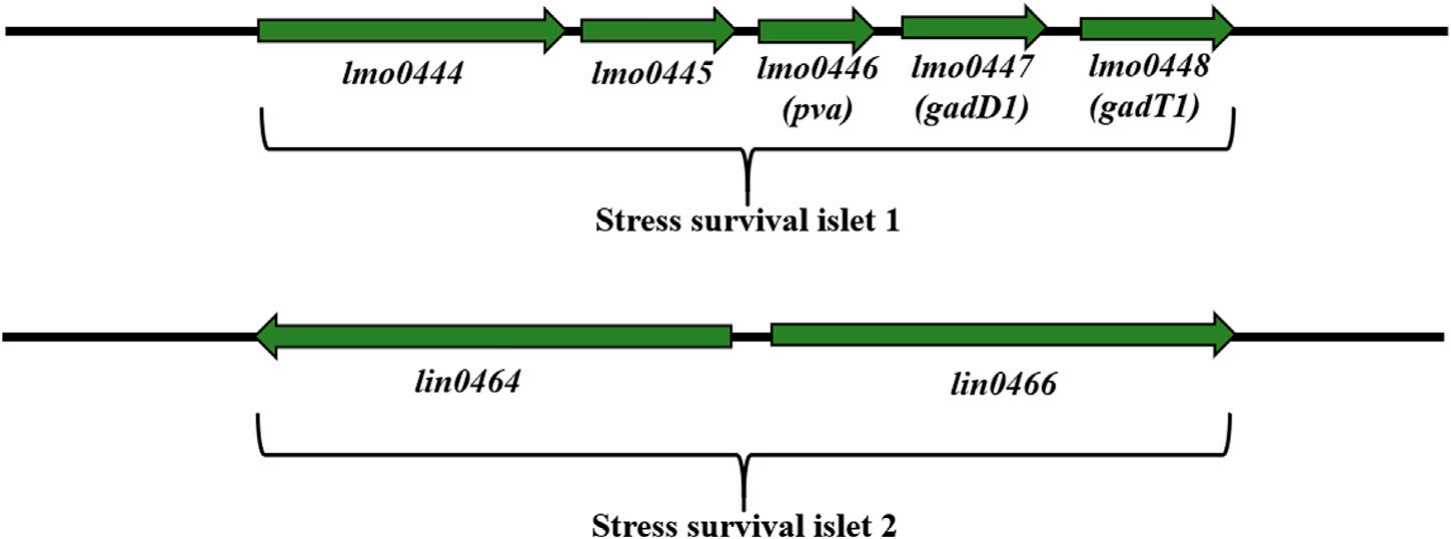
Organization of SSI-1 and SSI-2 among *L. monocytogenes* strains (according to: Ryan et al., 2009; Harter et al., 2017; Unrath et al., 2021). Direction of transcription is indicated by the respective arrows.

Stress survival islet (SSI-1) (8.7 kbp) is a region consisting of five genes: *lmo0444*, *lmo0464*, *pva* (*lmo0446*), *gadD1* (*lmo0447*) and *gadT1* (*lmo0448*) (Figure 6). These genes are associated with tolerance to acid, osmotic, and bile stress in the stomach (Begley et al., 2005; Cotter et al., 2005; Ryan et al., 2009; Begley et al., 2010) and are important for adaptation and survival in the food processing environment (Ryan et al., 2009). SSI-1 is located at the hypervariable region from *lmo0442* to *lmo0449* (Ryan et al., 2009). Lin0464 is a putative transcription regulator of the GntR family with a DNA helix-twist-helix binding domain. Lin0465 belongs to the DJ-1/PfpI protease superfamily with a type I glutamine amidotransferase-like domain characterized in *Pyrococcus furiosus* (Halio et al., 1998). Scientists have demonstrated the presence of SSI-1 among *L. monocytogenes* ST121 strains often isolated from the food processing environment (Hein et al., 2011; Schmitz-Esser et al., 2015; Rychli et al., 2017). Palacios-Gorba et al. (2021) have confirmed the presence of SSI-1 among *L. monocytogenes* strains (SL 1555) isolated from wild boar and deer tonsils. In turn, Liu et al. (Liu et al., 2022) have shown the presence of SSI-1 in *L. monocytogenes* ST5, ST121, and ST120, suggesting that these ST types may better tolerate the food processing environment than ST2. According to Hilliard et al. (2018), there is a relationship between the presence of SSI-1 and the persistence of *L. monocytogenes*. In addition, SSI-1 was strongly correlated with biofilm formation and truncated *inlA* gene (STOP codon) (Franciosa et al., 2009; Keeney et al., 2018; Lakicevic, Den Besten and De Biase, 2022). More recent findings confirmed the effect of SSI-1 and shortened *inlA* on increased biofilm production in *L. monocytogenes* (Lakicevic, Den Besten and De Biase, 2022). These data indicate the need for monitoring the environment and food processing area for virulent *L. monocytogenes* strains*.* Also, studies on the impact of SSI-1 on biofilm formation by *L. monocytogenes*under environmental stress conditions would be valuable.

#### Stress survival islet (SSI-2)

Harter et al. (2017) have documemted the presence of stress survival islet (SSI-2) mainly among *L. monocytogenes* ST121 strains (persisting for months and even years in food processing environments). SSI-2 consists of two genes: *lin0464* and *lin0465* (Figure 6), coding respectively, the transcription factor LIN0464 and the Pfpl protease beneficial for survival under alkaline and oxidative stress. SSI-2 is located at the hypervariable genetic point from *lmo0442* to *lmo0449*, which contains: SSI-1, SSI-2 and the homologue of the *LMOf2365_0481* gene (Harter et al., 2017). SSI-2 is highly conserved and occurs mainly in *L. monocytogenes* ST121 strains, the most abundant group of isolates in the food and food processing environment (Harter et al., 2017). Additionally, Harter et al. (2017) have shown that SSI-2 was not under σ^B^ control suggesting σ^H^ or σ^L^ regulation.

Interestingly, Harter et al. (2017) have noted that the presence of the *LMOf2365_0481* homologue is an alternative to SSI-2 and is common among clinical strains (function unknown). Gray et al. (2021) have found that the ST1 and ST2 isolates had the LMOf2365_0481 homologue.

The presence of SSI-2 may potentially support the adaptation and persistence of *L. monocytogenes* strains in the food processing environment. However, its role in the pathogens’ survival merits further investigation.

## Occurrence of genomic and pathogenicity islands among strains of *Listeria monocytogenes* isolated from various sources

Table 3 summarizes several studies on the frequency of genomic/pathogenicity islands among *L. monocytogenes* strains isolated from different sources in different regions worldwide. The LIPI-1 occurs in most *L. monocytogenes* strains, proving their virulence and ability to cause infection. The presence of SSI-1 and SSI-2 is typical for strains isolated from food and the food processing environment. Both SSI-1 and SSI-2 determine adaptation to stress conditions encountered during food processing. Also, LIPI-3 and LIPI-4 were detected in both environmental and clinical strains, which may indicate the hypervirulent nature of these strains. Nonetheless, researchers have reported a much higher frequency of LIPI-4 in clinical strains. On the contrary, LGI-1 island rarely occurs among strains of *L. monocytogenes*. There are also data on the occurrence of the recently discovered LGI-3 island among *L. monocytogenes* (Table 3). Monitoring the presence of virulence determinants in *L. monocytogenes* is of great importance.

## Transcription regulators of *L. monocytogenes*

L. *monocytogenes* possess four transcriptional regulators controlling virulence: PrfA, SigB, CodY, and VirR.

PrfA is the “major regulator of virulence” in *L. monocytogenes* because it directly activates all nine virulence genes (located on LIPI-1) and indirectly regulates more than 140 accessory genes (Milohanic et al., 2003; de las Heras et al., 2011). Other genes under the control of PrfA include internalins (involved in adhesion), the *hpt* gene (encodes a glucose-6-phosphate transport translocation involved in the proliferation of *L. monocytogenes* within the host cell cytoplasm) (Chico-Calero et al., 2002), and the *lapB* gene (encodes the surface protein LPXTG enabling adhesion into host cells) (Reis et al., 2010).

Most strains of *L. monocytogenes* have five sigma factors, including one main one - σ^A^ and four alternative ones - σ^B^, σ^C^, σ^H^, and σ^L^ (O’Byrne and Karatzas, 2008). σ^B^ regulates the expression of virulence genes in *L. monocytogenes* in response to environmental stress. The alternative factor sigma Bdirects the expression of over 300 genes involved in the stress response (NicAogain and O’Byrne, 2016). For the first time, Becker et al. (1998) demonstrated the role of σ^B^ in response to stress (osmotic). Further studies confirmed its participation in response to a variety of environmental conditions, including osmotic (Utratna et al., 2012), pH (Wiedmann et al., 1998; Wemekamp-Kamphuis et al., 2004), temperature (Liu et al., 2002) and oxidative stress (Ferreira et al., 2001).

σ^B^ interacts with PrfA in gene regulation, which is necessary for *L. monocytogenes* to achieve full virulence (Ollinger et al., 2009). Cross-talk between σ^B^ and PrfA ensures adequate gene expression both inside and outside the host (Gaballa et al., 2019). Interactions between σ^B^ and PrfA occur at the transcriptional, post-transcriptional, and translational levels (Gaballa et al., 2019). A direct connection exists through the σB-dependent monocistronic P2prfA promoter. In addition, the PrfA-regulated *inlAB* locus is also controlled by σB (Kazmierczak et al., 2003; Kim et al., 2005).

CodY is a transcriptional regulator that activates the transcription of *prfA* and other genes in response to low concentrations of branched-chain amino acids (BCAAs) found in host cells (Lobel et al., 2012; Lobel et al., 2015). Intracellular growth conditions also require adaptation to carbon uptake and nitrogen fixation from available sources. The nutrient-responsive regulator CodY coordinates *de novo* synthesis of BCAAs and sugar catabolism (Bennett et al., 2007; Lobel et al., 2012; Lobel et al., 2015). CodY represses the biosynthesis of amino acids (mainly BCAAs and histidine), purines, riboflavin, and some carbon and nitrogen metabolism genes under nutrient-rich conditions (Lemos et al., 2008; Pohl et al., 2009). Lobel and Herskovits (2016) showed that CodY activates the critical tricarboxylic acid (TCA) cycle enzymes, including glutamate/glutamine derivatives and the arginine biosynthesis pathway. Under nutrient-rich conditions, CodY directs metabolic flux from pyruvate to the TCA cycle via pyruvate carboxylase (PycA). At the same time, CodY directly represses the pyruvate oxidase gene (*poxB*, and the *ilv* operon), inhibiting pyruvate flux to the BCAA biosynthesis pathway (Lobel and Herskovits, 2016). CodY activates *prfA* transcription, by direct binding, in its coding sequence 15 nucleotides downstream of the start codon and thus stimulates the expression of virulence genes in response to low BCAA availability (Cossart and Archambaud, 2009). In response to nutrient excess CodY represses expression of σ^B^ and other genes (including: *sigB*, *arg*, *his*, *actA*, *glpF*, *gadG*, *gdhA*, *glnR* and *fla*) associated with metabolism, motility and virulence (Lobel and Herskovits, 2016).

Another but least characterized transcription regulator is VirR. VirR is a response regulator in a two-component system (TCS) consisting of a sensor histidine kinase and a response regulator (Mandin et al., 2005; Williams and Whitworth, 2010). The two-component VirR/S system controls the expression of 17 genes, including its own operon (Cossart, 2011) and the *dltABCD* operon (whose products are responsible for the incorporation of D-alanine into lipoteichoic acid) (Perego et al., 1995; Abachin et al., 2002). Also, the MprF protein (lysinylates phospholipids in the cell membrane of *L. monocytogenes*) (Thedieck et al., 2006) and AnrAB (an ATP-binding cassette (ABC) transporter) (Mandin et al., 2005; Collins et al., 2010) are under VirR control. According to Mandin et al. (2005), regulation of *dlt* and *mprF* by VirR suggests importance VirR/VirS system as a regulatorof *L. monocytogenes* resistance to human defensins or cationic peptides. Additionally, VirR is implicated in resistance to food preservatives and antimicrobials, especially for food preservation (Cossart, 2011; Kang et al., 2015). *L. monocytogenes* Δ*virR* mutants showed reduced virulence in a murine infection model (Cossart, 2011). The expression of VirR and many VirR-regulated genes arehighly induced during *in vivo* infection, suggesting that VirR participates in the detection of the host cell environment by *L. monocytogenes* (Cossart and Archambaud, 2009).

## Whole genome sequencing (WGS) for epidemiological purposes

### Whole genome sequencing (WGS) as a tool to search for new virulence determinants?

Whole genome sequencing (WGS) is a relevant tool in the virulence assessment of*L. monocytogenes*. This technique allowed the identification of five genomic/pathogenicity islands in *L. monocytogenes* (Table 4). The WGS also enables the characterization of strains responsible for outbreaks in the past when such modern techniques were not available, e.g., analysis of *L. monocytogenes*Scott A responsible for the listeriosis outbreak in Massachusetts (1983) (Briers et al., 2011).

**TABLE 4:** Genomic/pathogenic islands detected by Whole Genome Sequencing.

Strain	Characteristic	Identification of a new genomic island/pathogenicity	References
*L. monocytogenes* XYSN, 15LG, and 16E	- isolates from ovine listeriosis outbreaks	fragment of LIPI-2 containing genes: *smcL, i-inIF* and *i-inIE*	Yin et al. (2019)
	**-** members of a hybrid sub-lineage II (HSL-II), serotype 4h		
	- presence of LIPI-1, LIPI-3 and LIPI-4		
*L. monocytogenes*	CC4 clones	LIPI-4	Maury et al. (2016)
*L. monocytogenes* 08-5578 and 08-5923	- 1/2a serotype	LGI-1	Gilmour et al. (2010)
	- clinical isolates collected from individual outbreak-associated cases in Canada		
*L. monocytogenes* EGD-e	- 1/2a serotype	LGI-2	Kuenne et al. (2013)
	- lineage I		
*L. monocytogenes*	- persistent strains isolated in France	LGI-3	Palma et al. (2020)

LIPI, *Listeria* pathogenicity island; LGI, *Listeria* Genomic Island.

Constant analysis of the variability of *L. monocytogenes* strains isolated from various envi-ronments, combined with clinical, environmental, and epidemiological data, will allow us to approximate the selection mechanisms that can stimulate the adaptation of *L. monocytogenes* to different conditions (Disson et al., 2021). Therefore, it is essential to combine phenotypic and genotypic data to improve the value of the information provided by WGS (Wagner et al., 2022). Examples of such studies are the works of Stratakos et al. (2020), Kuenne et al. (2013), and Yan et al. (2019).

The WGS method has also been used in the identification of persistent *L. monocytogenes* strains (Stasiewicz et al., 2015; Cherifi et al., 2018; Wieczorek et al., 2020), which undoubtedly constitute a serious problem in the food industry.

### Whole genome sequencing (WGS) and epidemic

New features of*L. monocytogenes* strains, determining their hypervirulent nature, are constantly being identified. Quick strain identification may limit the pathogens’ spread, which is especially relevant during the outbreak. So far, the PFGE (Pulsed-field gel electrophoresis) method has been used as the gold standard (Ruppitsch et al., 2015; Centers for Disease Control and Prevention, 2016a; Neoh et al., 2019). In 2014, the implementation of whole genome sequencing under an epidemic study confirmed that this technique is arelevant tool for detecting outbreaks and quickly solving problems (Garner and Kathariou, 2016; Jackson et al., 2016; Moura et al., 2016; Pietzka et al., 2019). The WGS development has led to more accurate detection and analysis of the strains responsible for epidemics (Table 5). WGS allows the identification of single nucleotide polymorphisms (SNPs) and, thus, the determination of relatedness between different isolates (Moran-Gilad, 2017; Datta and Burall, 2018). Global surveillance of the food production system is critical in limiting the spread of microbial threats, including *L. monocytogenes*. WGS helped, among others, link the strains responsible for the outbreak in Australia with cases in Singapore (Das, 2019) or the US outbreak with cases in Australia (Kwong et al., 2016). In Europe, the WGSallowed the association of sporadic cases with food products, source attribution, and identification of the transmission pathway and antimicrobial resistance (Centers for Disease Control and Prevention, 2015a) (Table 5).

**TABLE 5:** The use of whole genome sequencing (WGS) for epidemiological purposes (chosen listeriosis outbreaks).

	Year	Location	Source	No. of cases (No. of deaths)	WGS analysis	References
United States	2014	Multistate	FNAO: mung bean sprouts	5 (2)	4b; high genetic relationship of mung bean sprout isolates, environmental isolates collected in the production plant with sequences of strains isolated from patients	Centers for Disease Control and Prevention (2015b)
	2014–2015	12 states	FNAO: caramel apples	35 (7)	- The WGS distinguished two isolates of *L. monocytogenes* (one in each cluster) with a high degree of similarity (PFGE - no distinction)	Centers for Disease Control and Prevention (2015a)
					- WGS analysis showed that *L. monocytogenes* isolates from whole apples produced by Bidart Bros collected along the distribution chain were strongly related to the outbreak strains	
					- It is estimated that the use of WGS shortened the epidemic by 1 week	
	2015	10 states	Soft cheeses distributed by Karoun dairies	30 (3)	- WGS gives a more detailed DNA fingerprint than PFGE	Centers for Disease Control and Prevention (2017a)
					- WGS - four different PFGE fingerprints were genetically closely related to the first PFGE fingerprint	
	2016	Two states	Raw milk	2 (1)	WGS - close genetic relationship of *L. monocytogenes* strains isolated from two patients and a milk sample	Centers for Disease Control and Prevention (2016b)
	2016	4 states	FNAO: frozen vegetables	9 (3)	The frozen corn isolate of *L. monocytogenes* was shown to be closely related to the eight patient isolates, and the frozen pea isolate to the patient isolate was shown to be closely related to the eight patient isolates	Centers for Disease Control and Prevention (2016c)
	2016	9 states	FNAO: packaged salads	19 (1)	- More fingerprint than PFGE	Centers for Disease Control and Prevention (2016d)
					- WGS analysis showed a close genetic relationship between isolates from patients in Canada and patient isolates in the United States	
	2017	4 states	Soft raw milk cheese	8 (2)	WGS analysis of patient isolates revealed a common source of *L. monocytogenes*	Centers for Disease Control and Prevention (2017b)
	2019	5 states	Hard-boiled eggs	8 (1)	The WGS showed that the bacteria in the environmental sample were genetically closely related to bacteria from sick people	Centers for Disease Control and Prevention (2020a)
	2020	4 states	Deli meats	12 (1)	Close genetic relationship of strains isolated from sick people	Centers for Disease Control and Prevention (2021)
	2022	11 states	Ice cream	28 (1)	WGS analysis showed that *L. monocytogenes* isolates collected from the ice cream and production environment were the source of the outbreak	Chen et al. (2017); Centers for Disease Control and Prevention (2022b)
	2022	8 states	FNAO: packaged salads	10 (1)	The WGS analysis enabled the detection of outbreak of *L. monocytogenes*	Centers for Disease Control and Prevention (2022c)
**Europe**	2015–2018	Austria, Denmark, Finland, Sweden, The United Kingdom	FNAO: frozen corn	41 (6)	The WGS analysis enabled the detection of a multi-country outbreak of *L. monocytogenes*	European Centre for Disease Prevention and Control (2018b)
	Since 2015	Denmark, Germany, France	RTE salmon products	12 (4)	The WGS analysis enabled the detection of a multi-country outbreak of *L. monocytogenes*	European Centre for Disease Prevention and Control (2018a)
	2014–2019	Denmark, Estonia, Finland, France, Sweden	Cold-smoked fish products	22 (5)	The WGS analysis enabled the detection of a multi-country outbreak of *L. monocytogenes*	European Centre for Disease Prevention and Control (2019b)
	2017–2019	Netherlands, Belgium	RTE meat products	21 (3)	The WGS analysis enabled the detection of a multi-country outbreak of *L. monocytogenes* (detected in wholesale and retail in four countries)	European Centre for Disease Prevention and Control (2019c)
	2019	Spain	Chilled roasted pork meat product	222 (3)	WGS analysis of *L. monocytogenes* isolates showed that human and food isolates share the same sequence	World Health Organization (2019)
Africa	2017–2018	Republic of South Africa	RTE processed meat products	1,024 (200)	- WGS analysis of isolates from a large subgroup of patients showed the same sequence type in a commonly consumed RTE processed meat product and in the manufacturer’s processing environment	NSW Department of Primary Industries (2018); World Health Organization (2018c)
					- All *L. monocytogenes* positives were further identified as the outbreak WGS strain belonging to MLST240	
Australia	2018	Australia (New South Wales, Victoria, Queensland, Tasmania)	FNAO: rockmelons	20 (7)	WGS analysis reported two cases in Singapore reported to be genetically linked to the Australian outbreak strain	Das (2019)

WGS, whole genome sequencing; PFGE, Pulsed-field gel electrophoresis; RTE, ready-to-eat; FNAO, Food of non-animal origin.

WGS is a helpful tool for strain genotype identification derived from different evolutionary lines and sources. When used for epidemiological purposes, WGS makes it possible to determine the source of *L. monocytogenes*.

## Conclusion

The genomic and pathogenicity islands are essential for the survival and adaption of *L. monocytogenes* to various environmental conditions. There are still many unknowns about the function of individual proteins and their role in the pathogenesis and virulence of *L. monocytogenes*, as well as the compilation of this information in relation to strains isolated from different environments. Understanding the genetics of *L. monocytogenes* is indispensable to controlling the pathogen and reducing the risk of listeriosis. Future studies should include a comparison of pheno- and genotypic characteristics to get a full picture of the virulence and ecology of *L. monocytogenes*. Research on the effect of stress factors on changes in phenotypic traits and consideration of horizontal gene transfer between *L. monocytogenes* strains and also other species present in the environment and food processing (especially antibiotic resistance genes) is desirable.
